# Calcium Orthophosphate-Based Bioceramics

**DOI:** 10.3390/ma6093840

**Published:** 2013-09-06

**Authors:** Sergey V. Dorozhkin

**Affiliations:** Kudrinskaja sq. 1-155, Moscow 123242, Russia; E-Mail: sedorozhkin@yandex.ru

**Keywords:** calcium orthophosphates, hydroxyapatite, tricalcium phosphate, bioceramics, biomaterials, grafts, biomedical applications, tissue engineering

## Abstract

Various types of grafts have been traditionally used to restore damaged bones. In the late 1960s, a strong interest was raised in studying ceramics as potential bone grafts due to their biomechanical properties. A bit later, such synthetic biomaterials were called bioceramics. In principle, bioceramics can be prepared from diverse materials but this review is limited to calcium orthophosphate-based formulations only, which possess the specific advantages due to the chemical similarity to mammalian bones and teeth. During the past 40 years, there have been a number of important achievements in this field. Namely, after the initial development of bioceramics that was just tolerated in the physiological environment, an emphasis was shifted towards the formulations able to form direct chemical bonds with the adjacent bones. Afterwards, by the structural and compositional controls, it became possible to choose whether the calcium orthophosphate-based implants remain biologically stable once incorporated into the skeletal structure or whether they were resorbed over time. At the turn of the millennium, a new concept of regenerative bioceramics was developed and such formulations became an integrated part of the tissue engineering approach. Now calcium orthophosphate scaffolds are designed to induce bone formation and vascularization. These scaffolds are often porous and harbor different biomolecules and/or cells. Therefore, current biomedical applications of calcium orthophosphate bioceramics include bone augmentations, artificial bone grafts, maxillofacial reconstruction, spinal fusion, periodontal disease repairs and bone fillers after tumor surgery. Perspective future applications comprise drug delivery and tissue engineering purposes because calcium orthophosphates appear to be promising carriers of growth factors, bioactive peptides and various types of cells.

## 1. Introduction

One of the most exciting and rewarding areas of the engineering discipline involves development of various devises for health care. Some of them are implantable. Examples comprise sutures, catheters, heart valves, pacemakers, breast implants, fracture fixation plates, nails and screws in orthopedics, various filling formulations, orthodontic wires, total joint replacement prostheses, *etc.* However, in order to be accepted by the living body without any unwanted side effects, all implantable items must be prepared from a special class of tolerable materials, called biomedical materials or biomaterials, in short. The physical character of the majority of the available biomaterials is solids [1,2].

Since all types of solids are divided into four major groups of materials: metals, polymers ceramics and various blends thereof, called composites, similarly, all types of solid biomaterials are also divided into the same groups: biometals, biopolymers, bioceramics and biocomposites. All of them play very important roles in both replacement and regeneration of human tissues; however, setting biometals, biopolymers and biocomposites aside, this review is focused on bioceramics only. In general, the modern bioceramics comprise polycrystalline materials, amorphous materials (glasses) and blends thereof (glass-ceramics). However, the chemical elements used to manufacture bioceramics form just a small set of the Periodic Table. Namely, bioceramics might be prepared from alumina, zirconia, magnesia, carbon, silica-contained and calcium-contained compounds, as well as from a limited number of other chemicals. All these formulations might be manufactured in both dense and porous forms in bulk, as well as in the forms of crystals, powders, particles, granules, scaffolds and/or coatings [1,2,3].

**Figure 1:**
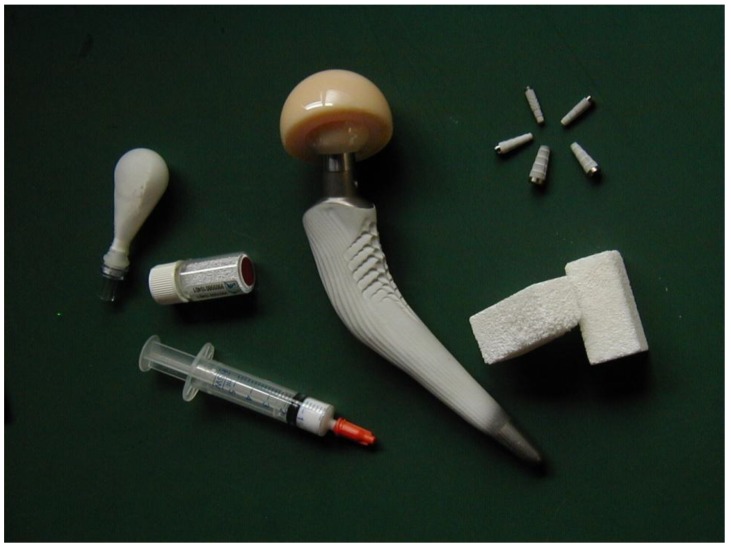
Several examples of the commercial calcium orthophosphate-based bioceramics.

As seen from the above, the entire subject of bioceramics is still rather broad. To specify it further, let me limit myself by a description of calcium orthophosphate-based bioceramics only. Due to the chemical similarity to mammalian bones and teeth, this type of bioceramics is used in a number of different applications throughout the body, covering all areas of the skeleton. The examples include healing of bone defects, fracture treatment, total joint replacement, bone augmentation, orthopedics, cranio-maxillofacial reconstruction, spinal surgery, otolaryngology, ophthalmology and percutaneous devices [1,2,3], as well as dental fillings and periodontal treatments [4]. Depending upon the required properties, different calcium orthophosphates might be used. For example, Figure 1 shows some randomly chosen samples of the commercially available calcium orthophosphate bioceramics for bone graft applications. One should note that, in 2010, only in the USA the sales of bone graft substitutes were valued at ~$1.3 billion with a forecast of ~$2.3 billion by 2017 [5]. This clearly demonstrates an importance of calcium orthophosphate-based bioceramics.

A list of the available calcium orthophosphates, including their standard abbreviations and major properties, is available in Table 1 [6]. To narrow the subject further, with a few important exceptions, bioceramics prepared from undoped and un-substituted calcium orthophosphates will be considered and discussed only. Due to this reason, calcium orthophosphate-based bioceramics prepared from biological resources, such as bones, teeth, corals, *etc.* [7,8,9,10,11,12,13,14,15,16,17,18,19,20,21,22,23,24,25], as well as the ion-substituted ones [26,27,28,29,30,31,32,33,34,35,36,37,38,39,40,41,42,43,44,45,46,47,48,49,50,51,52,53,54,55,56,57] is not considered. The readers interested in both topics are advised to study the original publications.

## 2. General Knowledge and Definitions

A number of definitions have been developed for the term “biomaterials”. For example, by the end of the 20th century, the consensus developed by the experts was the following: biomaterials were defined as synthetic or natural materials to be used to replace parts of a living system or to function in intimate contact with living tissues [58]. However, in September 2009, a more advanced definition was introduced: “A biomaterial is a substance that has been engineered to take a form which, alone or as part of a complex system, is used to direct, by control of interactions with components of living systems, the course of any therapeutic or diagnostic procedure, in human or veterinary medicine” [59]. The definition alterations were accompanied by a shift in both the conceptual ideas and the expectations of biological performance, which mutually changed in time [60].

In general, the biomaterials discipline is founded in the knowledge of the synergistic interaction of material science, biology, chemistry, medicine and mechanical science and it requires the input of comprehension from all these areas so that potential implants perform adequately in a living body and interrupt normal body functions as little as possible [61]. As biomaterials deal with all aspects of the material synthesis and processing, the knowledge in chemistry, material science and engineering is essential. On the other hand, as clinical implantology is the main purposes of biomaterials, biomedical sciences become the key part of the research. These include cell and molecular biology, histology, anatomy and physiology. The final aim is to achieve the correct biological interaction of the artificial grafts with living tissues of a host. In order to achieve the goals, several stages have to be performed, such as: material synthesis, design and manufacturing of prostheses, followed by various types of tests. Furthermore, any potential biomaterial must also pass all regulatory requirements before its clinical application [62].

**Table 1:** Existing calcium orthophosphates and their major properties [6].

Ca/P molar ratio	Compounds and their typical abbreviations	Chemical formula	Solubility at 25 °C, −log K_s_	Solubility at 25 °C, g/L	pH stability range in aqueous solutions at 25 °C
0.5	Monocalcium phosphate monohydrate (MCPM)	Ca(H_2_PO_4_)_2_·H_2_O	1.14	~18	0.0–2.0
0.5	Monocalcium phosphate anhydrous (MCPA or MCP)	Ca(H_2_PO_4_)_2_	1.14	~17	^[c]^
1.0	Dicalcium phosphate dihydrate (DCPD), mineral brushite	CaHPO_4_·2H_2_O	6.59	~0.088	2.0–6.0
1.0	Dicalcium phosphate anhydrous (DCPA or DCP), mineral monetite	CaHPO_4_	6.90	~0.048	^[c]^
1.33	Octacalcium phosphate (OCP)	Ca_8_(HPO_4_)_2_(PO_4_)_4_·5H_2_O	96.6	~0.0081	5.5–7.0
1.5	α-Tricalcium phosphate (α-TCP)	α-Ca_3_(PO_4_)_2_	25.5	~0.0025	^[a]^
1.5	β-Tricalcium phosphate (β-TCP)	β-Ca_3_(PO_4_)_2_	28.9	~0.0005	^[a]^
1.2–2.2	Amorphous calcium phosphates (ACP)	Ca*_x_*H*_y_*(PO_4_)*_z_*·*n*H_2_O, *n* = 3 − 4.5; 15%–20% H_2_O	^[b]^	^[b]^	~5–12 ^[d]^
1.5–1.67	Calcium-deficient hydroxyapatite (CDHA or Ca-def HA) ^[e]^	Ca_10−*x*_(HPO_4_)*_x_*(PO_4_)_6−*x*_(OH)_2−*x*_ (0 < *x* < 1)	~85	~0.0094	6.5–9.5
1.67	Hydroxyapatite (HA, HAp or OHAp)	Ca_10_(PO_4_)_6_(OH)_2_	116.8	~0.0003	9.5–12
1.67	Fluorapatite (FA or FAp)	Ca_10_(PO_4_)_6_F_2_	120.0	~0.0002	7–12
1.67	Oxyapatite (OA, OAp or OXA) ^[f]^	Ca_10_(PO_4_)_6_O	~69	~0.087	^[a]^
2.0	Tetracalcium phosphate (TTCP or TetCP), mineral hilgenstockite	Ca_4_(PO_4_)_2_O	38–44	~0.0007	^[a]^

^[a]^ These compounds cannot be precipitated from aqueous solutions; ^[b]^ Cannot be measured precisely. However, the following values were found: 25.7 ± 0.1 (pH = 7.40), 29.9 ± 0.1 (pH = 6.00), 32.7 ± 0.1 (pH = 5.28). The comparative extent of dissolution in acidic buffer is: ACP >> α-TCP >> β-TCP > CDHA >> HA > FA; ^[c]^ Stable at temperatures above 100 °C; ^[d]^ Always metastable; ^[e]^ Occasionally, it is called “precipitated HA (PHA)”; and ^[f]^ Existence of OA remains questionable.

In any case, biomaterials are intended to interface with biological systems *in vivo* to evaluate, treat, augment or replace any tissue, organ or function of the body and are now used in a number of different applications throughout the body. Thus, biomaterials are solely associated with the health care domain and must have an interface with tissues or tissue components. One should stress, that any artificial materials those simply are in contact with skin, such as hearing aids and wearable artificial limbs, are not included in the definition of biomaterials since the skin acts as a protective barrier between the body and the external world [1,2,63].

The major difference of biomaterials from other classes of materials lays in their ability to remain in a biological environment with neither damaging the surroundings nor being damaged in that process. Therefore, biomaterials must be distinguished from *biological materials* because the former are the materials that are accepted by living tissues and, therefore, they might be used for tissue replacements, while the latter are just the materials being produced by various biological systems (wood, cotton, bones, chitin, *etc.*) [64]. Furthermore, there are *biomimetic materials*, which are not made by living organisms but have the composition, structure and properties similar to those of biological materials. Concerning the subject of current review, *bioceramics* (or biomedical ceramics) is defined as biomaterials having the ceramic origin. Now it is important to define the meaning of ceramics. According to Wikipedia, the free encyclopedia: “The word ceramic comes from the Greek word κεραμικός (keramikos) meaning pottery, which is said to derive from the Indo-European word ker, meaning heat. A ceramic is an inorganic, non-metallic solid prepared by the action of heat and subsequent cooling. Ceramic materials may have a crystalline or partly crystalline structure, or may be amorphous (e.g., a glass). Because most common ceramics are crystalline, the definition of ceramic is often restricted to inorganic crystalline materials, as opposed to the non-crystalline glasses. Ceramic may be used as an adjective describing a material, product or process; or as a singular noun, or, more commonly, as a plural noun, ceramics.” [65]. As any other type of biomaterials, bioceramics can have structural functions as joint or tissue replacements, be used as coatings to improve the biocompatibility, as well as function as resorbable lattices, providing temporary structures and frameworks those are dissolved and/or replaced as the body rebuilds the damaged tissues [66,67,68,69,70,71]. Some types of bioceramics even feature a drug-delivery capability [72,73,74,75].

A progressive deterioration of all tissues with age is the major contributor to the need for spare parts for the body. Bones are especially vulnerable to fracture in older people due to a loss of density and strength with age. This effect is especially severe in women due to the hormonal changes associated with menopause. A graphical representation of the effect of time on bone strength and density from the age of 30 years onward is available in literature (Reference [68], Figure 1). Bone density decreases because bone-growing cells (osteoblasts) become progressively less productive in making new bone and repairing micro-fractures. The lower density greatly deteriorates the strength of bones and an unfortunate consequence is that many old people fracture their hips or have collapsed vertebrae and spinal problems [68].

In medicine, calcium orthophosphate bioceramics is needed to alleviate pain and restore functions of diseased or damaged calcified tissues (bones and teeth) of the body. A great challenge facing its medical application is, first, to replace and, second, to regenerate old and deteriorating bones with a biomaterial that can be replaced by a new mature bone without transient loss of a mechanical support [1,2]. Surface bioactivity is the major feature of calcium orthophosphate bioceramics. It contributes to a bone bonding ability and enhances new bone formation. After implantation, various interactions occur at the bioceramic/tissue interfaces leading to time-dependent changes in the surface characteristics of the implanted bioceramics and the surrounding tissues [76]. Because the average life span of humans is now 80+ years and the major need for spare parts begins at about 60 years of age, the after-effects of the implanted calcium orthophosphate bioceramics need to last, at least, for 20+ years. This demanding requirement of survivability is under conditions of use that are especially harsh to implanted biomaterials: corrosive saline solutions at 37 °C under variable, multiaxial and cyclical mechanical loads. The excellent performance of the specially designed calcium orthophosphate bioceramics that have survived these clinical conditions represented one of the most remarkable accomplishments of research, development, production and quality assurance by the end of the past century [68].

## 3. Bioceramics of Calcium Orthophosphates

### 3.1. History

The detailed history of hydroxyapatite and other calcium orthophosphates, including the subject of calcium orthophosphate bioceramics, as well as description of their past biomedical applications might be found elsewhere [77,78], where the interested readers are referred.

### 3.2. Chemical Composition and Preparation

Currently, calcium orthophosphate bioceramics can be prepared from various sources [7,8,9,10,11,12,13,14,15,16,17,18,19,20,21,22,23,24,25]. Nevertheless, up to now, all attempts to synthesize bone replacement materials for clinical applications featuring the physiological tolerance, biocompatibility and a long-term stability have had only a relative success; this clearly demonstrates both the superiority and a complexity of the natural structures [79].

In general, a characterization of calcium orthophosphate bioceramics should be performed from various viewpoints such as the chemical composition (including stoichiometry and purity), homogeneity, phase distribution, morphology, grain sizes and shape, grain boundaries, crystallite size, crystallinity, pores, cracks, surface roughness, *etc.* From the chemical point of view, the vast majority of calcium orthophosphate bioceramics is based on HA [80,81,82,83,84,85], both types of TCP [80,86,87,88,89,90,91,92,93,94,95] and/or multiphase formulations thereof. Biphasic formulations (commonly abbreviated as BCP—biphasic calcium phosphate) are the simplest among the latter ones. They include β-TCP + HA [96,97,98,99,100,101,102,103,104,105,106,107,108], α-TCP + HA [26,27,28,109,110] and biphasic TCP (commonly abbreviated as BTCP) consisting of α-TCP and β-TCP [111,112,113,114,115,116]. In addition, triphasic formulations (HA + α-TCP + β-TCP) have been prepared as well [117,118,119,120]. Further details on this topic might be found in a special review [121]. Leaving aside a big subject of DCPD-forming self-setting formulations [122,123], one should note that just a few publications on bioceramics, prepared from other types of calcium orthophosphates, are available [124,125,126,127,128,129,130].

The preparation techniques of various calcium orthophosphates have been extensively reviewed in literature [6,131,132,133,134,135,136] and references therein] where the interested readers are referred to. Briefly, when compared to both α- and β-TCP, HA is a more stable phase under the physiological conditions, as it has a lower solubility (Table 1) and, thus, a slower resorption kinetics [137,138,139,140]. Therefore, the BCP concept is determined by the optimum balance of a more stable phase of HA and a more soluble TCP. Due to a higher biodegradability of the α- or β-TCP component, the reactivity of BCP increases with the TCP/HA ratio increasing. Thus, *in vivo* bioresorbability of BCP can be controlled through the phase composition [101]. Similar conclusions are also valid for the biphasic TCP (in which α-TCP is a more soluble phase), as well as for both triphasic (HA, α-TCP and β-TCP) and yet more complex formulations.

**Figure 2:**
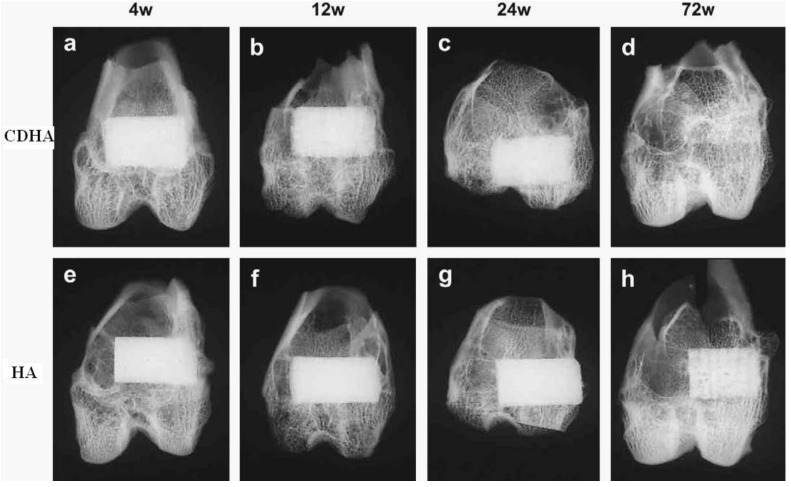
Soft X-ray photographs of the operated portion of the rabbit femur. (**a**) Four weeks; (**b**) 12 weeks; (**c**) 24 weeks; and (**d**) 72 weeks after implantation of CDHA. (**e**) Four weeks; (**f**) 12 weeks; (**g**)24 weeks; and (**h**) 72 weeks after implantation of sintered HA. Reprinted from Reference [141] with permission.

As implants made of sintered HA are found in bone defects for many years after implantation (Figure 2, bottom), bioceramics made of more soluble calcium orthophosphates [26,27,28,80,86,87,88,89,90,91,92,93,94,95,96,97,98,99,100,101,102,103,104,105,106,107,108,109,110,111,112,113,114,115,116,117,118,119,120,121,122,123,124,125,126,127,128,129,130,141,142,143] are preferable for the biomedical purposes (Figure 2, top). Furthermore, the experimental results showed that BCP had a higher ability to adsorb fibrinogen, insulin or type I collagen than HA [144]. Thus, according to both observed and measured bone formation parameters, calcium orthophosphate bioceramics have been ranked as follows: low sintering temperature BCP (rough and smooth) ≈ medium sintering temperature BCP ≈ TCP > calcined low sintering temperature HA > non-calcined low sintering temperature HA > high sintering temperature BCP (rough and smooth) > high sintering temperature HA [145]. This sequence has been developed in year 2000 and, thus, neither multiphase formulations, nor other calcium orthophosphates have been included.

### 3.3. Forming and Shaping

**Figure 3:**
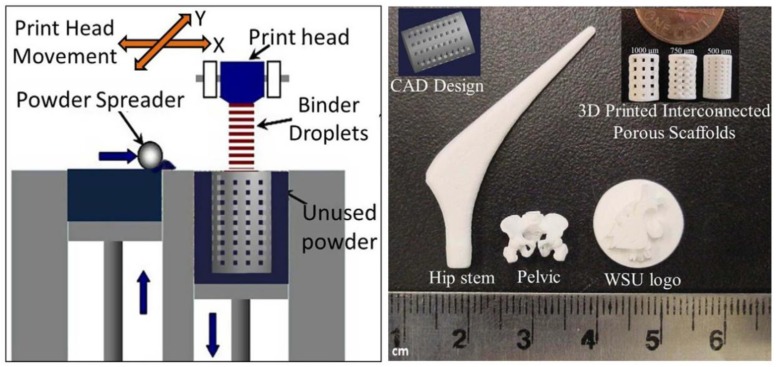
A schematic of 3D printing and some 3D printed parts (fabricated at Washington State University) showing the versatility of 3D printing technology for ceramic scaffolds fabrication with complex architectural features. Reprinted from Reference [74] with permission.

In order to fabricate bioceramics in progressively complex shapes, scientists are investigating the use of both old and new manufacturing techniques. These techniques range from an adaptation of the age-old pottery techniques to the newest manufacturing methods for high-temperature ceramic parts for airplane engines. Namely, reverse engineering [146,147] and rapid prototyping [148,149,150] technologies have revolutionized a generation of physical models, allowing the engineers to efficiently and accurately produce physical models and customized implants with high levels of geometric intricacy. Combined with the computer-aided design and manufacturing (CAD/CAM), complex physical objects of the anatomical structure can be fabricated in a variety of shapes and sizes. In a typical application, an image of a bone defect in a patient can be taken and used to develop a three-dimensional (3D) CAD computer model [151,152,153,154]. Then a computer can reduce the model to slices or layers. Afterwards, 3D objects and coatings are constructed layer-by-layer using rapid prototyping techniques. The examples comprise fused deposition modeling [155,156], selective laser sintering [157,158,159,160,161], laser cladding [162,163,164,165], 3D printing [91,166,167,168,169,170,171,172,173,174,175,176], solid freeform fabrication [177,178,179,180,181,182,183,184,185] and stereo lithography [186,187,188,189]. Furthermore, a thermal printing process of melted calcium orthophosphates has been proposed [190], while, in some cases, laser processing might be applied as well [191]. A schematic of 3D printing technique, as well as some 3D printed items are shown in Figure 3 [74]. A custom-made implant of actual dimensions would reduce the time it takes to perform the medical implantation procedure and subsequently lower the risk to the patient. Another advantage of a pre-fabricated, exact-fitting implant is that it can be used more effectively and applied directly to the damaged site rather than a replacement, which is formulated during surgery from a paste or granular material [178,191,192,193].

In addition to the aforementioned modern techniques, classical forming and shaping approaches are still widely used. The selection of the desired technique depends greatly on the ultimate application of the bioceramic device, e.g., whether it is for a hard-tissue replacement or an integration of the device within the surrounding tissues. In general, three types of the processing technologies might be used: (1) employment of a lubricant and a liquid binder with ceramic powders for shaping and subsequent firing; (2) application of self-setting and self-hardening properties of water-wet molded powders; (3) materials are melted to form a liquid and are shaped during cooling and solidification [194,195,196,197]. Since calcium orthophosphates are either thermally unstable (MCPM, MCPA, DCPA, DCPD, OCP, ACP, CDHA) or have a melting point at temperatures exceeding ~1400 °C with a partial decomposition (α-TCP, β-TCP, HA, FA, TTCP), only the first and the second consolidation approaches are used to prepare bulk bioceramics and scaffolds. The methods include uniaxial compaction [198,199,200], isostatic pressing (cold or hot) [108,201,202,203,204,205,206,207], granulation [208,209,210,211,212,213], loose packing [214], slip casting [93,215,216,217,218,219,220], gel casting [188,189,221,222,223,224,225], pressure mold forming [226], injection molding [227,228,229], polymer replication [230,231,232,233,234,235,236,237], extrusion [238,239,240,241,242], slurry dipping and spraying [243]. In addition, to form ceramic sheets from slurries, tape casting [105,223,244,245,246], doctor blade [247] and colander methods might be employed [194,195,196,197,248]. Various combinations of several techniques are also possible [95,223,249,250,251]. Furthermore, some of these processes might be performed under the electromagnetic field, which helps crystal aligning [216,220,252,253,254,255].

For example, powders are usually pressed damp in metal dies or dry in lubricated dies at pressures high enough to form sufficiently strong structures to hold together until they are sintered [256]. An organic binder such as polyvinyl alcohol helps to bind the powder together. The binder is removed by heating in air to oxidize the organic phases to carbon dioxide and water. Since many binders contain water, drying at ~100 °C is a critical step in preparing damp-formed pieces for firing. Too much or too little water in the compacts can lead to blowing apart the ware on heating or crumbling, respectively [194,195,196,197,202]. Furthermore, removal of water during drying often results in subsequent shrinkage of the product. In addition, due to local variations in water content, warping and even cracks may be developed during drying. Dry pressing and hydrostatic molding can minimize these problems [197]. Afterwards, the manufactured green samples are sintered.

Furthermore, forming and shaping of any ceramic products require a proper selection of the raw materials in terms of particle sizes and size distribution. Namely, tough and strong bioceramics consist of pure, fine and homogeneous microstructures. To attain this, pure powders with small average size and high surface area must be used as the starting sources. However, for maximum packing and least shrinkage after firing, mixing of ~70% coarse and ~30% fine powders have been suggested [197]. Mixing is usually carried out in a ball mill for uniformity of properties and reaction during subsequent firing. Mechanical die forming or sometimes extrusion through a die orifice can be used to produce a fixed cross-section.

Finally, to produce the accurate shaping, necessary for the fine design of bioceramics, machine finishing might be essential [153,194,257]. Unfortunately, cutting tools developed for metals are usually useless for bioceramics due to their fragility; therefore, grinding and polishing appear to be the convenient finishing techniques [153,194]. In addition, the surface of bioceramics might be modified by various supplementary treatments [258].

### 3.4. Sintering and Firing

A sintering (or firing) procedure appears to be of a great importance to manufacture bulk bioceramics with the required mechanical properties. Usually, this stage is carried out according to controlled temperature programs of electric furnaces in adjusted ambience of air with necessary additional gasses; however, always at temperatures below the melting points of the materials. The firing step can include temporary holds at intermediate temperatures to burn out organic binders [194,195,196,197]. The heating rate, sintering temperature and holding time depend on the starting materials. For example, in the case of HA, these values are in the ranges of 0.5–3 °C/min, 1000–1250 °C and 2–5 h, respectively [259]. In the majority cases, sintering allows a structure to retain its shape. However, this process might be accompanied by a considerable degree of shrinkage [260,261,262], which must be accommodated in the fabrication process. For instance, in the case of FA sintering, a linear shrinkage was found to occur at ~715 °C and the material reached its final density at ~890 °C. Above this value, grain growth became important and induced an intra-granular porosity, which was responsible for density decrease. At ~1180 °C, a liquid phase was formed due to formation of a binary eutectic between FA and fluorite contained in the powder as impurity. This liquid phase further promoted the coarsening process and induced formation of large pores at high temperatures [263].

**Figure 4:**
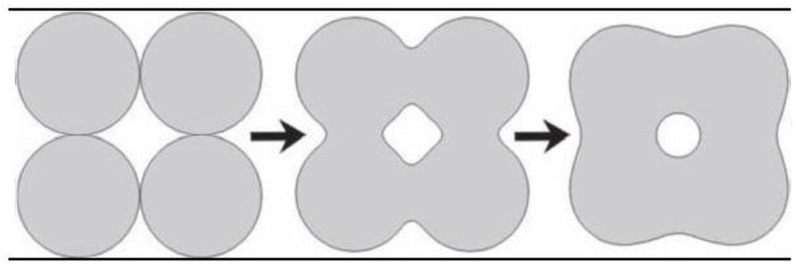
A schematic diagram representing the changes occurring with particles under sintering. Shrinkage is noticeable.

In general, sintering occurs only when the driving force is sufficiently high, while the latter relates to the decrease in surface and interfacial energies of the system by matter (molecules, atoms or ions) transport, which can proceed by solid, liquid or gaseous phase diffusion. Namely, when solids are heated to high temperatures, their constituents are driven to move to fill up pores and open channels between the grains of powders, as well as to compensate for the surface energy differences among their convex and concave surfaces (matter moves from convex to concave). At the initial stages, bottlenecks are formed and grow among the particles (Figure 4). Existing vacancies tend to flow away from the surfaces of sharply curved necks; this is an equivalent of a material flow towards the necks, which grow as the voids shrink. Small contact areas among the particles expand and, at the same time, a density of the compact increases and the total void volume decreases. As the pores and open channels are closed during a heat treatment, the particles become tightly bonded together and density, strength and fatigue resistance of the sintered object improve greatly. Grain-boundary diffusion was identified as the dominant mechanism for densification [264]. Furthermore, strong chemical bonds are formed among the particles and loosely compacted green bodies are hardened to denser materials [194,195,196,197]. Further knowledge on the ceramic sintering process might be found elsewhere [265].

**Figure 5:**
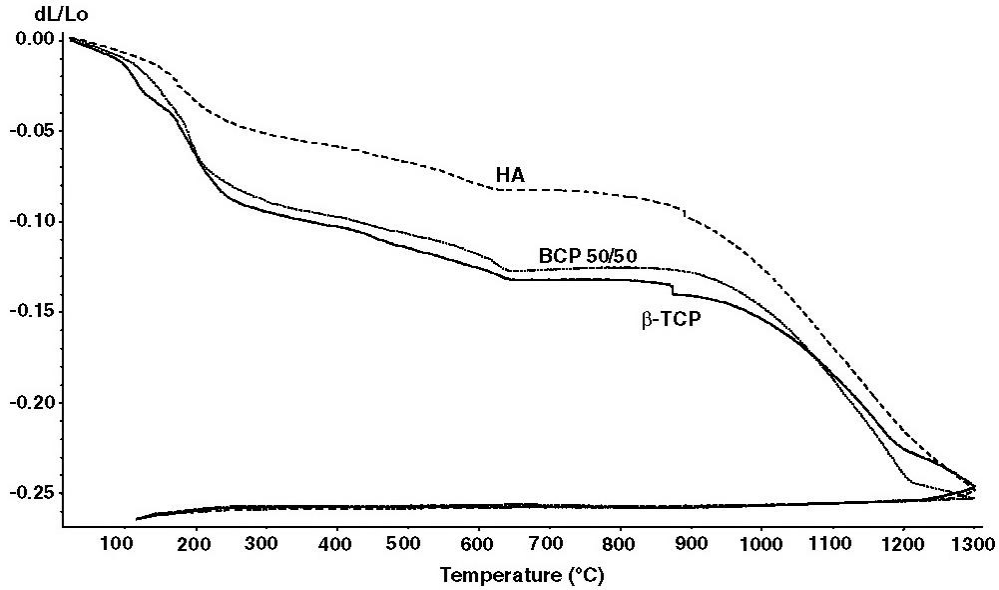
Linear shrinkage of the compacted ACP powders that were converted into β-TCP, BCP (50% HA + 50% β-TCP) and HA upon heating. According to the authors: “At 1300 °C, the shrinkage reached a maximum of approximately ~25%, ~30% and ~35% for the compacted ACP powders that converted into HA, BCP 50/50 and β-TCP, respectively” [261]. Reprinted from Reference [261] with permission.

In the case of calcium orthophosphates, the earliest paper on their sintering was published in 1971 [266]. Since then, numerous papers on this subject were published and several specific processes were found to occur during calcium orthophosphate sintering. Firstly, moisture, carbonates and all other volatile chemicals remaining from the synthesis stage, such as ammonia, nitrates and any organic compounds, are removed as gaseous products. Secondly, unless powders are sintered, the removal of these gases facilitates production of denser ceramics with subsequent shrinkage of the samples (Figure 5). Thirdly, all chemical changes are accompanied by a concurrent increase in crystal size and a decrease in the specific surface area. Fourthly, a chemical decomposition of all acidic orthophosphates and their transformation into other phosphates (e.g., 2HPO_4_^2−^ → P_2_O_7_^4−^ + H_2_O↑) takes place. Besides, sintering causes toughening [84], densification [85,267], partial dehydroxylation (in the case of HA) [85], grain growth [264,268], as well as it increases the mechanical strength [269,270,271]. The latter events are due to presence of air and other gases filling gaps among the particles of un-sintered powders. At sintering, the gases move towards the outside of powders and green bodies shrink owing to decrease of distances among the particles. For example, sintering of a biologically formed apatites was investigated [272,273] and the obtained products were characterized [274,275]. In all cases, the numerical value of Ca/P ratio in sintered apatites of biological origin was higher than that of the stoichiometric HA. One should mention that in the vast majority cases, calcium orthophosphates with Ca/P ratio <1.5 (Table 1) are not sintered, since these compounds are thermally unstable, while sintering of non-stoichiometric calcium orthophosphates (CDHA and ACP) always leads to their transformation into various types of biphasic, triphasic and multiphase formulations [121].

An extensive study on the effects of sintering temperature and time on the properties of HA bioceramics revealed a correlation between these parameters and density, porosity, grain size, chemical composition and strength of the scaffolds [276]. Namely, sintering below ~1000 °C was found to result in initial particle coalescence, with little or no densification and a significant loss of the surface area and porosity. The degree of densification appeared to depend on the sintering temperature whereas the degree of ionic diffusion was governed by the period of sintering [276]. To enhance sinterability of calcium orthophosphates, a variety of sintering additives might be added [277,278,279,280].

Solid-state pressureless sintering is the simplest procedure. For example, HA bioceramics can be pressurelessly sintered up to the theoretical density at 1000–1200 °C. Processing at even higher temperatures usually lead to exaggerated grain growth and decomposition because HA becomes unstable at temperatures exceeding ~1300 °C [6,131,132,133,134,135,136,281,282,283,284]. The decomposition temperature of HA bioceramics is a function of the partial pressure of water vapor. Moreover, processing under vacuum leads to an earlier decomposition of HA, while processing under high partial pressure of water prevents from the decomposition. On the other hand, a presence of water in the sintering atmosphere was reported to inhibit densification of HA and accelerated grain growth [248,285]. Unexpectedly, an application of a magnetic field during sintering was found to influence the growth of HA grains [268]. A definite correlation between hardness, density and a grain size in sintered HA bioceramics was found: despite exhibiting high bulk density, hardness started to decrease at a certain critical grain size limit [286,287,288].

Since grain growth occurs mainly during the final stage of sintering, to avoid this, a new method called ‘‘two-step sintering’’ (TSS) was proposed [289]. The method consists of suppressing grain boundary migration responsible for grain growth, while keeping grain boundary diffusion that promotes densification. The TSS approach was successfully applied to calcium orthophosphate bioceramics [107,290,291,292,293]. For example, HA compacts prepared from nanodimensional powders were two-step sintered. The average grain size of near full dense (>98%) HA bioceramics made via conventional sintering was found to be ~1.7 μm, while that for TSS HA bioceramics was ~190 nm (*i.e.*, ~9 times less) with simultaneous increasing the fracture toughness of samples from 0.98 ± 0.12 to 1.92 ± 0.20 MPa m^1/2^. In addition, due to the lower second step sintering temperature, no HA phase decomposition was detected in TSS method [290].

Hot pressing [288,294,295,296,297,298,299], hot isostatic pressing [108,201,206] or hot pressing with post-sintering [300,301] processes make it possible to decrease a temperature of the densification process, diminish the grain size, as well as achieve higher densities. This leads to finer microstructures, higher thermal stability and subsequently better mechanical properties of calcium orthophosphate bioceramics. Microwave [18,302,303,304,305,306,307,308,309,310] and spark plasma [87,124,311,312,313,314,315,316,317,318,319,320,321] sintering techniques are alternative methods to the conventional sintering, hot pressing and hot isostatic pressing. Both alternative methods were found to be time and energy efficient densification techniques. Further developments are still possible. For example, a hydrothermal hot pressing method has been developed to fabricate OCP [125], CDHA [322], HA/β-TCP [296] and HA [297,298,300,323] bioceramics with neither thermal dehydration nor thermal decomposition. Further details on the sintering and firing processes of calcium orthophosphate bioceramics are available in literature [67,133,248,324,325].

To conclude this part, one should mention that the sintering stage is not always necessary. For example, calcium orthophosphate-based bulk bioceramics with the reasonable mechanical properties might be prepared by means of self-setting (self-hardening) formulations (see Section 5.1 below). Furthermore, the reader’s attention is paid on an excellent review on various ceramic manufacturing techniques [326], in which various ceramic processing techniques are well described.

## 4. The Major Properties

### 4.1. Mechanical Properties

The modern generation of biomedical materials should stimulate the body’s own self-repairing abilities [327]. Therefore, during healing, a mature bone should replace the modern grafts and this process must occur without transient loss of the mechanical support. Unluckily for material scientists, a human body provides one of the most inhospitable environments for the implanted biomaterials. It is warm, wet and both chemically and biologically active. For example, a diversity of body fluids in various tissues might have a solution pH varying from 1 to 9. In addition, a body is capable of generating quite massive force concentrations and the variance in such characteristics among individuals might be enormous. Typically, bones are subjected to ~4 MPa, whereas tendons and ligaments experience peak stresses in the range of 40–80 MPa. The hip joints are subjected to an average load up to three times body weight (3000 N) and peak loads experienced during jumping can be as high as 10 times body weight. These stresses are repetitive and fluctuating depending on the nature of the activities, which can include standing, sitting, jogging, stretching and climbing. Therefore, all types of implants must sustain attacks of a great variety of aggressive conditions [328]. Regrettably, there is presently no artificial material fulfilling all these requirements.

Now it is important to mention, that the mechanical behavior of any ceramics is rather specific. Namely, ceramics is brittle, which is attributed to high strength ionic bonds. Thus, it is not possible for plastic deformation to happen prior to failure, as a slip cannot occur. Therefore, ceramics fail in a dramatic manner. Namely, if a crack is initiated, its progress will not be hindered by the deformation of material ahead of the crack, as would be the case in a ductile material (e.g., a metal). In ceramics, the crack will continue to propagate, rapidly resulting in a catastrophic breakdown [195]. All of these are applicable to calcium orthophosphate bioceramics.

For dense bioceramics, the strength is a function of the grain sizes. It appears to be very sensitive to a slow crack growth [329]. Finer grain size bioceramics have smaller flaws at the grain boundaries and thus are stronger than one with larger grain sizes. In general, the mechanical properties decrease significantly with increasing content of an amorphous phase, microporosity and grain sizes, while a high crystallinity, a low porosity and small grain sizes tend to give a higher stiffness, a higher compressive and tensile strength and a greater fracture toughness. Accordingly, from the mechanical point of view, calcium orthophosphate bioceramics appear to be brittle polycrystalline materials for which the mechanical properties are governed by crystallinity, grain size, grain boundaries, porosity and composition [207]. Thus, it possesses poor mechanical properties (for instance, a low impact and fracture resistances) that do not allow calcium orthophosphate bioceramics to be used in load-bearing areas, such as artificial teeth or bones [66,67,68,69,70,71,330]. For example, fracture toughness (this is a property, which describes the ability of a material containing a crack to resist fracture and is one of the most important properties of any material for virtually all design applications) of HA bioceramics does not exceed the value of ~1.2 MPa·m^1/2^ [331] (human bone: 2–12 MPa·m^1/2^). It decreases almost linearly with a porosity increasing [248]. Generally, fracture toughness increases with grain size decreasing. However, in some materials, especially non-cubic ceramics, fracture toughness reaches the maximum and rapidly drops with decreasing grain size. For example, a fracture toughness of pure hot pressed HA with grain sizes between 0.2–1.2 µm was investigated. The authors found two distinct trends, where fracture toughness decreased with increasing grain size above ~0.4 µm and subsequently decreased with decreasing grain size. The maximum fracture toughness measured was 1.20 ± 0.05 MPa·m^1/2^ at ~0.4 µm [299]. Fracture energy of HA bioceramics is in the range of 2.3–20 J/m^2^, while the Weibull modulus (it is a measure of the spread or scatter in fracture strength) is low (~5–12) in wet environments, which means that HA behaves as a typical brittle ceramics and indicates to a low reliability of HA implants [248]. Porosity has a great influence on the Weibull modulus [332,333]. Interestingly that 3 peaks of internal friction were found at temperatures about −40, 80 and 130 °C for HA but no internal friction peaks were obtained for FA in the measured temperature range; this effect was attributed to the differences of F^−^ and OH^−^ positions in FA and HA, respectively [334]. The differences in internal friction values were also found between HA and TCP [335].

Bending, compressive and tensile strengths of dense HA bioceramics are in the ranges of 38–250 MPa, 120–900 MPa and 38–300 MPa, respectively. Similar values for porous HA bioceramics are substantially lower: 2–11 MPa, 2–100 MPa and ~3 MPa, respectively [248]. These wide variations in the properties are due to both structural variations (e.g., an influence of remaining microporosity, grain sizes, presence of impurities, *etc.*) and manufacturing processes, as well as they are caused by a statistical nature of the strength distribution. Strength was found to increase with Ca/P ratio increasing, reaching the maximum value around Ca/P ~1.67 (stoichiometric HA) and decreases suddenly when Ca/P > 1.67 [248]. Furthermore, strength decreases almost exponentially with porosity increasing [96,97]. However, by changing the pore geometry, it is possible to influence the strength of porous bioceramics. It is also worth mentioning that porous HA bioceramics is considerably less fatigue resistant than dense ones (in materials science, fatigue is the progressive and localized structural damage that occurs when a material is subjected to cyclic loading). Both grain sizes and porosity are reported to influence the fracture path, which itself has a little effect on the fracture toughness of calcium orthophosphate bioceramics [207,336]. However, no obvious decrease in mechanical properties was found after calcium orthophosphate bioceramics had been aged in the various solutions during the different periods of time [337].

Young’s (or elastic) modulus of dense HA bioceramics is in the range of 35–120 GPa [338,339], which is more or less similar to those of the most resistant components of the natural calcified tissues (dental enamel: ~74 GPa, dentine: ~21 GPa, compact bone: ~18–22 GPa). This value depends on porosity [340]. Nevertheless, dense bulk compacts of HA have mechanical resistances of the order of 100 MPa *vs.* ~300 MPa of human bones, diminishing drastically their resistances in the case of porous bulk compacts [341]. Young’s modulus measured in bending is between 44 and 88 GPa. To investigate the subject in more details, various types of modeling and calculations are increasingly used [342,343,344,345,346]. For example, the elastic properties of HA appeared to be significantly affected by the presence of vacancies, which softened HA via reducing its elastic modules [346]. In addition, a considerable anisotropy in the stress-strain behavior of the perfect HA crystals was found by *ab initio* calculations [343]. The crystals appeared to be brittle for tension along the *z*-axis with the maximum stress of ~9.6 GPa at 10% strain. Furthermore, the structural analysis of the HA crystal under various stages of tensile strain revealed that the deformation behavior manifested itself mainly in the rotation of PO_4_ tetrahedrons with concomitant movements of both the columnar and axial Ca ions [343]. Data for single crystals are also available [347]. Vickers hardness (that is a measure of the resistance to permanent indentation) of dense HA bioceramics is within 3–7 GPa, while the Poisson’s ratio (that is the ratio of the contraction or transverse strain to the extension or axial strain) for HA is about 0.27, which is close to that of bones (~0.3). At temperatures within 1000–1100 °C, dense HA bioceramics was found to exhibit superplasticity with a deformation mechanism based on grain boundary sliding [321,348,349]. Furthermore, both wear resistance and friction coefficient of dense HA bioceramics are comparable to those of dental enamel [248].

Due to a high brittleness (associated to a low crack resistance), the biomedical applications of calcium orthophosphate bioceramics are focused on production of non-load-bearing implants, such as pieces for middle ear surgery, filling of bone defects in oral or orthopedic surgery, as well as coating of dental implants and metallic prosthesis (see below) [79,350,351]. Therefore, ways are continuously sought to improve the reliability of calcium orthophosphate bioceramics. Namely, the mechanical properties of sintered bioceramics might be improved by changing the morphology of the initial calcium orthophosphates [352]. In addition, diverse reinforcements (ceramics, metals or polymers) have been applied to manufacture various biocomposites and hybrid biomaterials [353], but that is another story. However, successful hybrid formulations consisted of calcium orthophosphates only [354,355,356,357,358,359,360,361] are within the scope of this review. Namely, bulk HA bioceramics might be reinforced by HA whiskers [355,356,357,358,359]. Furthermore, various biphasic apatite/TCP formulations were tested [354,360,361] and, for example, a superior superplasticity of HA/β-TCP biocomposites to HA bioceramics was detected [360].

Another approach to improve the mechanical properties of calcium orthophosphate bioceramics is to cover the items by polymeric coatings [362,363,364] or infiltrate porous structures by polymers [365]; however, this is still other story. Further details on the mechanical properties of calcium orthophosphate bioceramics are available elsewhere [248,366], where the interested readers are referred to.

### 4.2. Electrical Properties

Occasionally, an interest to the electrical properties of calcium orthophosphates is expressed [306,367,368,369,370]. For example, a surface ionic conductivity of both porous and dense HA bioceramics was examined for humidity sensor applications, since the room temperature conductivity was influenced by relative humidity [371]. Namely, the ionic conductivity of HA has been a subject of research for its possible use as a gas sensor for alcohol [372], carbon dioxide [372,373] or carbon monoxide [374]. Electrical measurements have also been used as a characterization tool to study the evolution of microstructure in HA bioceramics [375]. More to the point, Valdes *et al.* examined the dielectric properties of HA to understand its decomposition to β-TCP [376]. In the case of CDHA, the electrical properties, in terms of ionic conductivity, were found to increase after compression of the samples at 15 t/cm^2^, which was attributed to establishment of some order within the apatitic network [377]. The conductivity mechanism of CDHA appeared to be multiple [378]. Furthermore, there was an attempt to develop CDHA whisker electrets for biomedical utilization [379].

Interestingly that the electrical properties of calcium orthophosphate bioceramics appear to influence their biomedical applications. For example, there is an interest in polarization of HA bioceramics to generate a surface charge by the application of electric fields at elevated temperatures [380,381]. The presence of surface charges on HA was shown to have a significant effect on both *in vitro* and *in vivo* crystallization of biological apatite [382,383,384,385,386,387,388]. Furthermore, a growth of both biomimetic calcium orthophosphates and bones was found to be accelerated on negatively charged surfaces and decelerated at positively charged surfaces [386,387,388,389,390,391,392,393,394,395,396,397,398,399]. Similar effect was found for adsorption of bovine serum albumin [400]. In addition, the electrical polarization of calcium orthophosphates was found to accelerate a cytoskeleton reorganization of osteoblast-like cells [401,402,403,404], extend bioactivity [405] and enhance bone ingrowth through the pores of porous implants [406]. The positive effect of electric polarization was found for carbonated apatite as well [407]. There is an interesting study on the interaction of a blood coagulation factor on electrically polarized HA surfaces [408]. Further details on the electrical properties of calcium orthophosphate-based bioceramics are available in literature [306,409,410,411,412,413].

### 4.3. Possible Transparency

**Figure 6:**
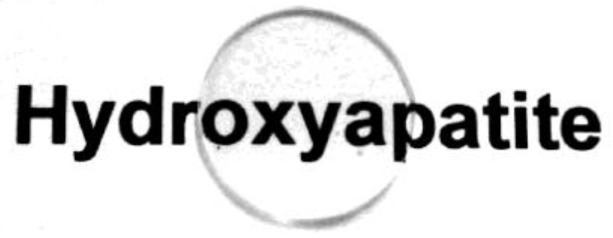
Transparent HA bioceramics prepared by spark plasma sintering at 900 °C from nano-sized HA single crystals. Reprinted from Reference [414] with permission.

Single crystals of all calcium orthophosphates are optically transparent for the visible light. As bioceramics of calcium orthophosphates have a polycrystalline nature with a random orientation of big amounts of small crystals, it is opaque and of white color, unless colored dopants have been added. However, in some cases, a transparency is convenient to provide some essential advantages (e.g., to enable direct viewing of living cells, their attachment, spreading, proliferation, and osteogenic differentiation cascade in a transmitted light). Thus, transparent calcium orthophosphate bioceramics (Figure 6) [414] have been prepared and investigated [87,108,201,203,317,319,320,321,414,415,416,417,418,419,420,421,422,423,424]. They can exhibit an optical transmittance of ~66% at a wavelength of 645 nm [423]. The preparation techniques include a hot isostatic pressing [108,201,203], an ambient-pressure sintering [415], gel casting coupled with a low-temperature sintering [418,421], a pulse electric current sintering [419], as well as a spark plasma sintering [87,317,318,319,320,321]. Fully dense, transparent calcium orthophosphate bioceramics are obtained at temperatures above ~800 °C. Depending on the preparation technique, the transparent bioceramics has a uniform grain sizes ranging from ~67 nm [108] to ~250 μm [418] and always is pore-free. Furthermore, a translucent calcium orthophosphate bioceramics is also known [108,266,425,426,427]. However, due to a lack of both porosity and the necessity to have see-through implants inside the body, the transparent and translucent forms of calcium orthophosphate bioceramics will hardly be ever used in medicine with the specific eye implants as the only reasonable exception.

### 4.4. Porosity

**Figure 7:**
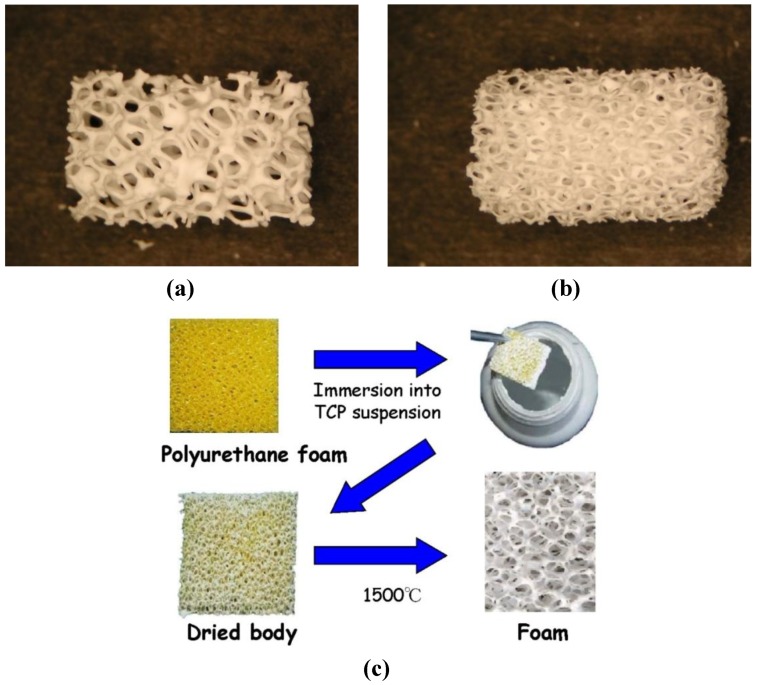
Photographs of a commercially available porous calcium orthophosphate bioceramics with (**a,b**) different porosity; and (**c**) a method of their production. For photos, the horizontal field width is 20 mm. The picture (**c**) is reprinted from Reference [457] with permission.

Porosity is defined as a percentage of voids in solids and this morphological property is independent of the material. The surface area of porous bodies is much higher, which guarantees a good mechanical fixation in addition to providing sites on the surface that allow chemical bonding between the bioceramics and bones [428]. Furthermore, a porous material may have both closed (isolated) pores and open (interconnected) pores. The latter look like tunnels and are accessible by gases, liquids and particulate suspensions [429]. The open-cell nature of porous materials (also known as reticulated materials) is a unique characteristic essential in many applications. In addition, pore dimensions are also important. Namely, the dimensions of open pores are directly related to bone formation, since such pores grant both the surface and space for cell adhesion and bone ingrowth [430,431]. On the other hand, pore interconnection provides the ways for cell distribution and migration, as well as it allows an efficient *in vivo* blood vessel formation suitable for sustaining bone tissue neo-formation and possibly remodeling [144,432,433,434,435,436,437,438,439,440,441,442]. Namely, porous calcium orthophosphate bioceramics can be colonized by bone tissues [437,442,443,444,445,446,447,448,449,450,451,452,453,454]. Therefore, interconnecting macroporosity (pore size >100 μm) [104,428,437,455,456], which is defined by its capacity to be colonized by cells, is intentionally introduced in solid bioceramics (Figure 7).

**Figure 8:**
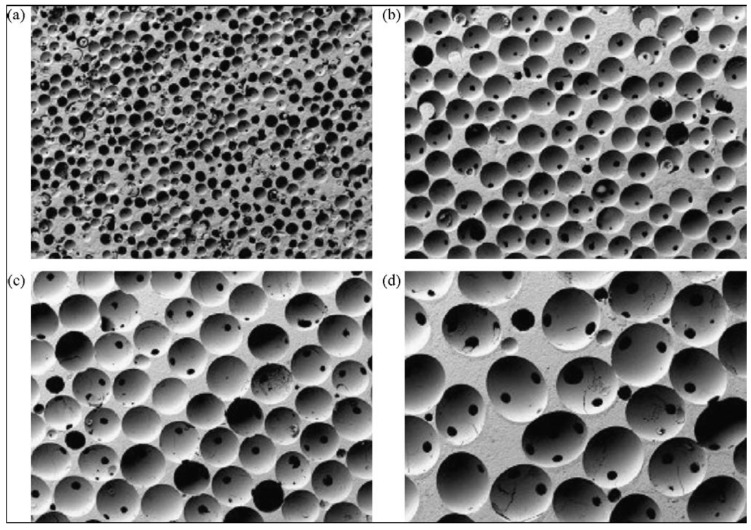
β-TCP porous ceramics with different pore sizes prepared using polymethylmethacrylate balls with diameter equal to: (**a**) 100–200 μm; (**b**) 300–400 μm; (**c**) 500–600 μm; and (**d**) 700–800 μm. Horizontal field width is 45 mm. Reprinted from Reference [92] with permission.

Macroporosity is usually formed due to a release of various easily removable compounds and, for that reason, incorporation of pore-creating additives (porogens) is the most popular technique to create macroporosity. The porogens are crystals, particles or fibers of either volatile (they evolve gases at elevated temperatures) or soluble substances, such as paraffin [458,459,460], naphthalene [207,461,462,463], sucrose [464,465], NaHCO_3_ [466,467,468], NaCl [469,470], polymethylmethacrylate [92,471,472,473], hydrogen peroxide [474,475,476,477,478] and cellulose derivatives [82]. Several other compounds [96,181,324,479,480,481,482,483,484,485,486,487,488,489,490] might be used as porogens, as well. Obviously, the ideal porogen should be nontoxic and be removed at ambient temperature, thereby allowing the bioceramic/porogen mixture to be injected directly into a defect site and allowing the scaffold to fit the defect [491]. Sintering particles, preferably spheres of equal size, is a similar way to generate porous 3D bioceramics of calcium orthophosphates (Figure 8). However, pores resulting from this method are often irregular in size and shape and not fully interconnected with one another.

Many other techniques, such as replication of polymer foams by impregnation [230,231,232,235,492,493,494,495] (Figure 7), various types of casting [217,219,223,225,496,497,498,499,500,501,502,503,504,505], suspension foaming [120], surfactant washing [506], as well as several other approaches [86,89,92,161,507,508,509,510,511,512,513,514,515,516,517,518,519,520,521,522,523,524,525,526,527,528,529,530,531,532,533,534,535,536,537,538,539,540,541,542,543,544,545,546,547,548] have been applied to fabricate porous calcium orthophosphate bioceramics. Some of them have been summarized in Table 2 [491]. In addition, natural porous materials, as coral skeletons [549,550,551] or shells [552] made of CaCO_3_, can be converted into porous calcium orthophosphates under the hydrothermal conditions (250 °C, 24–48 h) with the microstructure undamaged. Porous HA bioceramics can also be obtained by hydrothermal hot pressing. This technique allows solidification of the HA powder at 100–300 °C (30 MPa, 2 h) [323]. In another approach, bi-continuous water-filled microemulsions have been used as pre-organized systems for the fabrication of needle-like frameworks of crystalline HA (2 °C, 3 weeks) [510,511]. Besides, porous calcium orthophosphates might be prepared by a combination of gel casting and foam burn out methods [249,251], as well as by setting of the self-setting formulations [459,460,467,468,470,479,480,543]. Lithography was used to print a polymeric material, followed by packing with HA and sintering [514]. Both hot pressing [294,295] and ice templating [515,516,517,518] techniques were applied as well. More to the point, a HA suspension can be cast into a porous CaCO_3_ skeleton, which is then dissolved, leaving a porous network [508]. 3D periodic macroporous frame of HA has been fabricated via a template-assisted colloidal processing technique [519,520]. More to the point, porous HA bioceramics might be prepared by using different starting HA powders and sintering at various temperatures by a pressureless sintering [523]. Porous bioceramics with an improved strength might be fabricated from calcium orthophosphate fibers or whiskers. In general, fibrous porous materials are known to exhibit an improved strength due to fiber interlocking, crack deflection and/or pullout [553]. Namely, porous bioceramics with well-controlled open pores was processed by sintering of fibrous HA particles [512]. In another approach, porosity was achieved by firing apatite-fiber compacts mixed with carbon beads and agar. By varying the compaction pressure, firing temperature and carbon/HA ratio, the total porosity was controlled in the ranges from ~40% to ~85% [82]. Finally, a superporous (~85% porosity) HA bioceramics was developed as well [540,541,542]. Additional information on the processing routes to produce porous ceramics might be found in literature [554].

**Figure 9:**
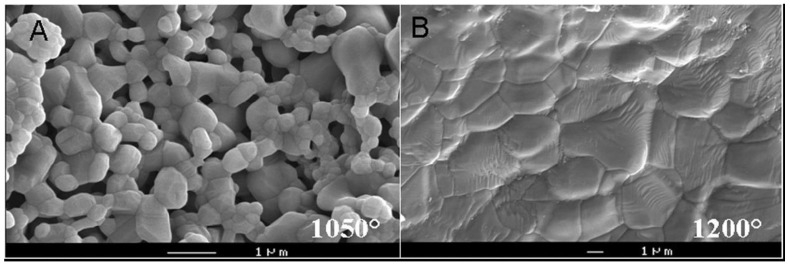
SEM pictures of HA bioceramics sintered at **(A)** 1050 °C; and **(B)** 1200 °C. Note the presence of microporosity in (**A**) and not in (**B**). Scale bar is 1 μm. Reprinted from Reference [563] with permission.

Bioceramic microporosity (pore size <10 μm), which is defined by its capacity to be impregnated by biological fluids [555], results from the sintering process, while the pore dimensions mainly depend on the material composition, thermal cycle and sintering time. The microporosity provides both a greater surface area for protein adsorption and increased ionic solubility. For example, embedded osteocytes distributed throughout microporous rods might form a mechanosensory network, which would not be possible in scaffolds without microporosity [556,557]. Calcium orthophosphate bioceramics with nanodimensional (<100 nm) pores might be fabricated as well [196,558,559,560,561,562]. It is important to stress, that differences in porogens usually influence the bioceramics’ macroporosity, while differences in sintering temperatures and conditions affect the percentage of microporosity. Usually, the higher the sintering temperature, the lower both the microporosity content and the specific surface area of bioceramics. Namely, HA bioceramics sintered at ~1200 °C shows significantly less microporosity and a dramatic change in crystal sizes, if compared with that sintered at ~1050 °C (Figure 9) [563]. Furthermore, the average shape of pores was found to transform from strongly oblate to round at higher sintering temperatures [564]. The total porosity (macroporosity + microporosity) of calcium orthophosphate bioceramics was reported to be ~70% [565] or even ~85% [540,541,542] of the entire volume. In the case of coralline HA or bovine-derived apatites, the porosity of the original biologic material (coral or bovine bone) is usually preserved during processing [566]. To finalize the production topic, creation of the desired porosity in calcium orthophosphate bioceramics is a rather complicated engineering task and the interested readers are referred to additional publications on the subject [96,433,567,568,569,570,571,572,573,574,575,576,577,578,579].

**Table 2:** The procedures used to manufacture porous calcium orthophosphate scaffolds for tissue engineering [491].

Year	Location	Process	Apatite from:	Sintering	Compressive strength	Pore size	Porosity	Method of porosity control
2006	Deville *et al.* Berkeley, CA	HA + ammonium methacrylate in PTFE mold, freeze dried and sintered	HA #30	Yes: 1300 °C	16 MPa 65 MPa 145 MPa	open unidirectional 50–150 μm	>60% 56% 47%	Porosity control: slurry conc. Structure controlled by physics of ice front formation.
2006	Saiz *et al.* Berkeley, CA	Polymer foams coated, compressed after infiltration, then calcined.	HA powder	Yes: 700–1300 °C	–	100–200 μm	–	Porosity control: extent of compression, HA loading
2006	Murugan *et al.* Singapore + USA	Bovine bone cleaned, calcined	bovine bone	Yes: 500 °C	–	retention of nano-sized pores	–	Porosity control: native porosity of bovine bone
2006	Xu *et al.* Gaithersburg, MD	Directly injectable calcium orthophosphate cement, self hardens, mannitol as porogen	nanocrystalline HA	No	2.2–4.2 MPa (flexural)	0%–50% macroporous	65%–82%	Porosity control: mannitol mass fraction in mixture
2004	Landi *et al.* Italy + Indonesia	Sponge impregnation, isotactic pressing, sintering of HA in simulated body fluid	CaO + H_3_PO_4_	Yes: 1250 °C for 1 h	23 ± 3.8 MPa	closed 6% open 60%	66%	Porosity control: possibly by controlling HA particle size. Not suggested by authors
2003	Charriere *et al.* EPFL, Switzerland	Thermoplastic negative porosity by Ink jet printing, slip casting process for HA	DCPA + calcite	No: 90 °C for 1 day	12.5 ± 4.6 MPa	–	44%	Porosity control: negative printing
2003	Almirall *et al.* Barcelona, Spain	α-TCP foamed with hydrogen peroxide at different conc., liq. ratios, poured in PTFE molds	α-TCP + (10% and 20% H_2_O_2_)	No: 60 °C for 2 h	1.41 ± 0.27 MPa 2.69 ± 0.91 MPa	35.7% macro 29.7% micro 26.8% macro 33.8% micro	65.5% 60.7%	Porosity control: different concentration, α-TCP particle sizes
2003	Ramay *et al.* Seattle, WA	Slurries of HA prepared: gel-casting + polymer sponge technique, sintered.	HA powder	Yes: 600 °C for 1 h 1350 °C for 2 h	0.5–5 MPa	200–400 μm	70%–77%	Porosity control: replicate of polymer sponge template
2003	Miao *et al.* Singapore	TTCP to calcium orthophosphate cement cement. Slurry cast on polymer foam, sintered.	TTCP	Yes: 1200 °C for 2 h	–	1 mm macro 5 μm micro	~70%	Porosity control: Recoating time, polyurethane foam
2003	Uemura *et al.* China + Japan	Slurry of HA with polyoxyethylenelaurylether (cross-linked) and sintered	HA powders	Yes: 1200 °C for 3 h	2.25 MPa (0 wk) 4.92 MPa (12 wks) 11.2 MPa (24 wks)	500 μm 200 μm interconnects	~77%	Porosity control: polymer interconnects cross-linking
2003	Ma *et al.* Singapore + USA	Electrophoretic deposition of HA, sintering.	HA powders	Yes: 1200 °C for 2 h	860 MPa	0.5 μm 130 μm	~20%	Porosity control: electrophoresis field
2002	Barralet *et al.* Birmingham, London, UK	Calcium orthophosphate cement + sodium phosphate ice, evaporated	CaCO_3_ + DCPD	1st step: 1400 °C for 1 day	0.6 ± 0.27 MPa	2 μm	62% ± 9%	Porosity control: porogen shape.

Concerning the biomedical importance of porosity, studies revealed that increasing of both the specific surface area and pore volume of bioceramics might greatly accelerate the *in vivo* process of apatite deposition and, therefore, enhance the bone-forming bioactivity. More importantly, a precise control over the porosity, pore dimensions and internal pore architecture of bioceramics on different length scales is essential for understanding of the structure-bioactivity relationship and the rational design of better bone-forming biomaterials [576,580,581]. Namely, in antibiotic charging experiments, a calcium orthophosphate bioceramics with nanodimensional (<100 nm) pores showed a much higher charging capacity (1621 μg/g) than that of commercially available calcium orthophosphate (100 μg/g), which did not contain nanodimensional porosity [572]. In other experiments, porous blocks of HA were found to be viable carriers with sustained release profiles for drugs [582] and antibiotics over 12 days [583] and 12 weeks [584], respectively. Unfortunately, porosity significantly decreases the strength of implants [248,336,366]. Thus, porous calcium orthophosphate implants cannot be loaded and are used to fill only small bone defects. However, their strength increases gradually when bones ingrow into the porous network of calcium orthophosphate implants [139,585,586,587,588]. For example, Martin *et al.* reported bending strengths of 40–60 MPa for a porous HA implant filled with 50%–60% of cortical bone [585], while in another study an ingrown bone increased strength of porous HA bioceramics by a factor of 3 to 4 [587].

Unfortunately, the biomedical effects of bioceramics’ porosity are not straightforward. For example, the *in vivo* response of calcium orthophosphates of different porosity was investigated and a hardly any effect of macropore dimensions (~150, ~260, ~510 and ~1220 μm) was observed [589]. In another study, a greater differentiation of mesenchymal stem cells was observed when cultured on ~200 μm pore size HA scaffolds when compared to those on ~500 μm pore size HA [590]. The latter finding was attributed to the fact that a higher pore volume in ~500 μm macropore scaffolds might contribute to a lack of cell confluency leading to the cells proliferating before beginning differentiation. Besides, the authors hypothesized that bioceramics having a less than the optimal pore dimensions induced quiescence in differentiated osteoblasts due to reduced cell confluency [590]. In still another study, the use of BCP (HA/TCP = 65/35 wt. %) scaffolds with cubic pores of ~500 μm resulted in the highest bone formation compared with the scaffold with lower (~100 μm) or higher (~1000 μm) pore sizes [591]. Furthermore, calcium orthophosphate bioceramics with greater strut porosity appeared to be more osteoinductive [592]. Already in 1979, Holmes suggested that the optimal pore range was 200–400 μm with the average human osteon size of ~223 μm [550]. In 1997, Tsurga and coworkers implied that the optimal pore size of bioceramics that supported ectopic bone formation was 300–400 μm [593]. Thus, there is no need to create calcium orthophosphate bioceramics with very big pores; however, the pores must be interconnected [440,455,456,594]. Interconnectivity governs a depth of cells or tissue penetration into the porous bioceramics, as well as it allows development of blood vessels required for new bone nourishing and wastes removal [555,595]. Nevertheless, the total porosity of implanted bioceramics appears to be important. For example, 60% porous β-TCP granules achieved a higher bone fusion rate than 75% porous β-TCP granules in lumbar posterolateral fusion [556].

## 5. Biomedical Applications

Since Levitt *et al.* described a method of preparing a FA bioceramics and suggested its possible use in medical applications in 1969 [596], calcium orthophosphate bioceramics have been widely tested for clinical applications. Namely, a great number of forms, compositions and trade-marks (Table 3) currently are either in use or under a consideration in many areas of orthopedics and dentistry, with even more in development. For example, bulk materials, available in dense and porous forms, are used for alveolar ridge augmentation, immediate tooth replacement and maxillofacial reconstruction [4]. Other examples comprise burr-hole buttons [597,598], orbital implants (including Bio-Eye^®^) [599,600,601,602,603,604,605,606], increment of the hearing ossicles [607,608,609], spine fusion [610,611,612,613] and repair of bone defects [138,614,615]. In order to permit growth of new bone into defects, a suitable bioresorbable material should fill these defects. Otherwise, ingrowth of fibrous tissue might prevent bone formation within the defects.

**Table 3:** Registered commercial trademarks (current and past) of calcium orthophosphate-based bioceramics and biomaterials.

Calcium orthophosphate	Trade name and producer (when available)
CDHA	Calcibon (Biomet, IN, USA)
	Cementek (Teknimed, France)
	Osteogen (Impladent, NY, USA)
HA	Actifuse (ApaTech, UK)
	Alveograf (Cooke-Waite Laboratories, USA)
	Apaceram (HOYA Corp., PENTAX New Ceramics Division, Japan)
	ApaPore (ApaTech, UK)
	Bio-Eye (Integrated Orbital Implants, CA, USA)
	BioGraft (IFGL BIO CERAMICS, India)
	Bioroc (Depuy-Bioland, France)
	Bonefil (Pentax, Japan)
	Bonetite (Pentax, Japan)
	Boneceram (Sumitomo Osaka Cement, Japan)
	BoneSource (Stryker Orthopaedics, NJ, USA)
	Calcitite (Zimmer, IN, USA)
	Cerapatite (Ceraver, France)
	Durapatite (unknown producer)
	HA BIOCER (CHEMA–ELEKTROMET, Poland)
	HA^nan^° Surface (Promimic, Sweden)
	nanoXIM (Fluidinova, Portugal)
	Neobone (Covalent Materials, Japan)
	OssaBase-HA (Lasak, Czech Republic)
	Ostegraf (Ceramed, CO, USA)
	Ostim (Heraeus Kulzer, Germany)
	Periograf (Cooke-Waite Laboratories, USA)
	PermaOS (Mathys, Switzerland)
	PurAtite (PremierBiomaterials, Ireland)
	Synatite (SBM, France)
	Synthacer (KARL STORZ Recon, Germany)
	without trade name (Cam Bioceramics, Netherlands)
	without trade name (CaP Biomaterials, WI, USA)
HA embedded in silica gel	NanoBone (Artoss, Germany)
HA/collagen	Bioimplant (Connectbiopharm, Russia)
	Bonject (Koken, Japan)
	Collagraft (Zimmer and Collagen Corporation, USA)
	CollapAn (Intermedapatite, Russia)
	HAPCOL (Polystom, Russia)
	LitAr (LitAr, Russia)
HA/sodium alginate	Bialgin (Biomed, Russia)
HA/poly-L-Lactic Acid	SuperFIXSORB30 (Takiron, Japan)
HA/polyethylene	HAPEX (Gyrus, TN, USA)
HA/CaSO_4_	Hapset (LifeCore, MIN, USA)
	PerOssal (aap Implantate, Germany)
coralline HA	Interpore (Interpore, CA, USA)
	ProOsteon (Interpore, CA, USA)
algae-derived HA	Algipore (Dentsply Friadent, Germany)
bovine bone apatite (unsintered)	BioOss (Geitslich, Switzerland)
	Laddec (Ost-Developpement, France)
	Lubboc (Ost-Developpement, France)
	Oxbone (Bioland biomateriaux, France)
	Tutoplast (Tutogen Medical, Germany)
bovine bone apatite (sintered)	BonAP (unknown producer)
	Cerabone (aap Implantate, Germany)
	Endobon (Merck, Germany)
	Navigraft (Zimmer Dental, USA)
	Osteograf (Ceramed, CO, USA)
	PepGen P-15 (Dentsply Friadent, Germany)
	Pyrost (Osteo AG, Germany)
hyman bone allograft	maxgraft (botiss, Germany)
	Osnatal (aap Implantate, Germany)
equine	BioGen (unknown producer)
α-TCP	BioBase (Zimmer, IN, USA)
	without trade name (Cam Bioceramics, Netherlands)
	without trade name (PremierBiomaterials, Ireland)
β-TCP	adboneTCP (Medbone Medical Devices, Portugal)
	Antartik TCP (MedicalBiomat, France)
	Augment Bone Graft (BioMimetic Therapeutics, TN, USA)
	BioGraft (IFGL BIO CERAMICS, India)
	Bioresorb (Sybron Implant Solutions, Germany)
	Biosorb (SBM S.A., France)
	Bi-Ostetic (Berkeley Advanced Biomaterials, CA, USA)
	Calc-i-oss classic (Degradable Solutions, Switzerland)
	Calciresorb (Ceraver, France)
	CELLPLEX (Wright Medical Technology, TN, USA)
	Cerasorb (Curasan, Germany)
	Ceros (Thommen Medical, Switzerland)
	ChronOS (Synthes, PA, USA)
	Conduit (DePuy Spine, USA)
	GenerOs (Berkeley Advanced Biomaterials, CA, USA)
	HT BIOCER (CHEMA–ELEKTROMET, Poland)
	JAX (Smith and Nephew Orthopaedics, USA)
	Osferion (Olympus Terumo Biomaterials, Japan)
	OsSatura TCP (Integra Orthobiologics, CA, USA)
	PORESORB-TCP (Lasak, Czech Republic)
	SynthoGraft (Bicon, MA, USA)
	Synthos (unknown producer)
	Syntricer (KARL STORZ Recon, Germany)
	Vitoss (Orthovita, PA, USA)
	without trade name (Cam Bioceramics, Netherlands)
	without trade name (CaP Biomaterials, WI, USA)
	without trade name (Shanghai Bio-lu Biomaterials, China)
β-TCP/collagen	Integra Mozaik (Integra Orthobiologics, CA, USA)
BCP (HA + β-TCP)	4Bone (MIS, Israel)
	adboneBCP (Medbone Medical Devices, Portugal)
	Antartik Genta (MedicalBiomat, France)
	Artosal (aap Implantate, Germany)
	BCP BiCalPhos (Medtronic, MN, USA)
	BioGraft (IFGL BIO CERAMICS, India)
	Biosel (Depuy Bioland, France)
	BonaGraft (Biotech One, Taiwan)
	BoneCeramic (Straumann, Switzerland)
	BoneSave (Stryker Orthopaedics, NJ, USA)
	Calcicoat (Zimmer, IN, USA)
	Calciresorb (Ceraver, France)
	Calc-i-oss crystal (Degradable Solutions, Switzerland)
	CellCeram (Scaffdex, Finland)
	Ceraform (Teknimed, France)
	Ceratite (NGK Spark Plug, Japan)
	CuriOs (Progentix Orthobiology BV, Netherlands)
	Eurocer (FH Orthopedics, France)
	GenPhos HA TCP (Baumer, Brazil)
	Graftys BCP (Graftys, France)
	Hatric (Arthrex, Naples, FL, USA)
	Hydros (Biomatlante, France)
	Indost (Polystom, Russia)
	Kainos (Signus, Germany)
	MasterGraft Granules (Medtronic Sofamor Danek, TN, USA)
	MBCP (Biomatlante, France)
	OrthoCer HA TCP (Baumer, Brazil)
	OpteMX (Exactech, FL, USA)
	OsSatura BCP (Integra Orthobiologics, CA, USA)
	ossceram nano (bredent medical, Germany)
	Osteosynt (Einco, Brazil)
	Ostilit (Stryker Orthopaedics, NJ, USA)
	ReproBone (Ceramisys, UK)
	SBS (Expanscience, France)
	Scaffdex (Scaffdex Oy, Finland)
	TCH (Kasios, France)
	Triosite (Zimmer, IN, USA)
	Tribone (Stryker, Europe)
	without trade name (Cam Bioceramics, Netherlands)
	without trade name (CaP Biomaterials, WI, USA)
BCP (HA + α-TCP)	Skelite (Millennium Biologix, ON, Canada)
BCP (HA + β-TCP)/collagen	Allograft (Zimmer, IN, USA)
	Collagraft (Zimmer, IN, USA)
BCP/fibrin	TricOS (Baxter BioScience, France)
BCP/silicon	FlexHA (Xomed, FL, USA)
FA	without trade name (CaP Biomaterials, WI, USA)
FA + BCP (HA + β-TCP)	FtAP (Polystom, Russia)
carbonateapatite	Healos (Orquest, CA, USA)
	SRS (Norian, CA, USA)

**Figure 10:**
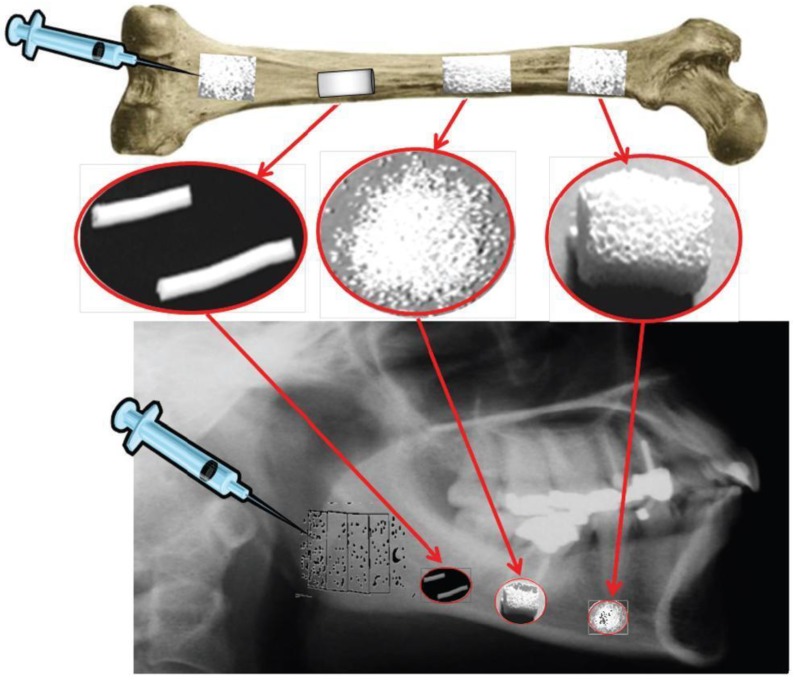
Different types of biomedical applications of calcium orthophosphate bioceramics. Reprinted from Reference [623] with permission.

In spite of the aforementioned serious mechanical limitations (see Section 4.1), bioceramics of calcium orthophosphates is available in various physical forms: powders, particles, granules (or granulates [12]), dense blocks, porous scaffolds, self-setting formulations, implant coatings and composite component of different origin (natural, biological or synthetic) often with the specific shapes, such as implants, prostheses or prosthetic devices. Furthermore, bone grafts are also proposed as non-hardening injectable formulations [99,616,617,618,619] and pastes [620,621,622]. Generally, they consist of a mixture of calcium orthophosphate powders or granules and a “glue”, which can be a highly viscous hydrogel [99,619]. More to the point, custom-designed shapes like wedges for tibial opening osteotomy, cones for spine and knee and inserts for vertebral cage fusion are also available [565]. Various trademarks of the commercially available types of calcium orthophosphate-based bioceramics and biomaterials have been summarized in Table 3, while their surgical applications are schematically shown in Figure 10 [623]. A long list of both trademarks and producers clearly demonstrates that calcium orthophosphate bioceramics is easy to produce and not very difficult to register for the biomedical applications.

One should note, that among the existing calcium orthophosphates (Table 1), only certain compounds are useful for biomedical applications, because those having the Ca/P ionic ratio less than 1 are not suitable for implantation due to their high solubility and acidity. Furthermore, due to its basicity, TTCP alone is not suitable either. However, to be used in medicine, these “unsuitable” calcium orthophosphates might be successfully combined with either other calcium orthophosphates or other chemicals.

### 5.1. Self-Setting (Self-Hardening) Formulations

The need for bioceramics for minimal invasive surgery has induced a concept of self-setting (or self-hardening) formulations consisting of calcium orthophosphates only to be applied as injectable and/or mouldable bone substitutes [122,123,145,480,514,624,625,626,627,628]. In addition, there are reinforced formulations, which, in a certain sense, might be defined as calcium orthophosphate concretes [122]. Furthermore, self-setting formulations able to form porous bioceramics are also available [459,460,467,468,470,479,480,514,543,625,626,627,628].

**Figure 11:**
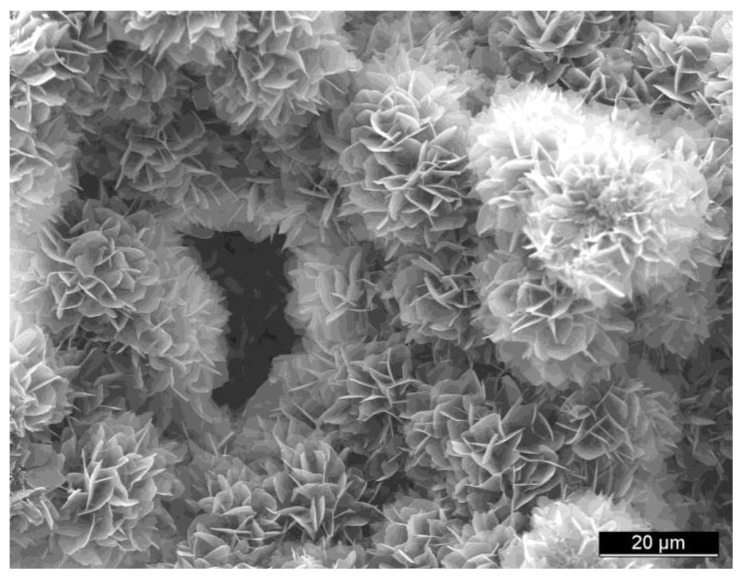
A typical microstructure of calcium orthophosphate cement after hardening. The mechanical stability is provided by the physical entanglement of crystals. Reprinted from Reference [629] with permission.

All types of the self-setting calcium orthophosphate formulations belong to a low temperature bioceramics. They are divided into two major groups. The first one is a dry mixture of two different calcium orthophosphates (a basic one and an acidic one), in which, after being wetted, the setting reaction occurs according to an acid-base reaction. The second group contains only one calcium orthophosphate, such as ACP with Ca/P molar ratio within 1.50–1.67 or α-TCP: both of them form CDHA upon contact with an aqueous solution [122,145]. Chemically, setting (=hardening, curing) is due to the succession of dissolution and precipitation reactions. Mechanically, it results from crystal entanglement and intergrowth (Figure 11) [629]. Despite a large number of the initial compositions, all types of self-setting calcium orthophosphate formulations can form two different products only: CDHA and DCPD [122,123,145,480,514,624,625,626,627,628]. Special reviews on the subject are available [122], where the interested readers are referred for further details.

### 5.2. Coatings, Films and Layers

For many years, the clinical application of calcium orthophosphate-based bioceramics has been largely limited to non-load bearing parts of the skeleton due to their inferior mechanical properties. Therefore, materials with better mechanical properties appear to be necessary. For example, metallic implants are encountered in endoprostheses (total hip joint replacements) and artificial teeth sockets. As metals do not undergo bone bonding, *i.e.*, do not form a mechanically stable link between the implant and bone tissue, ways have been sought to improve contacts at the interface. The major way is to coat metals with calcium orthophosphates those exhibit a bone-bonding ability between the metal and bone [194,206,385,630,631,632,633,634].

**Figure 12:**
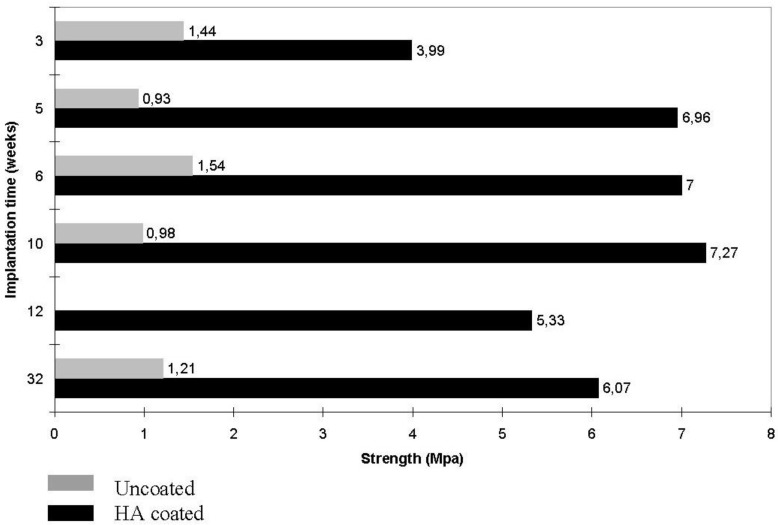
Shows how a plasma-sprayed HA coating on a porous titanium (dark bars) dependent on the implantation time will improve the interfacial bond strength compared to uncoated porous titanium (light bars). Reprinted from Reference [66] with permission.

A number of factors influence the properties of calcium orthophosphate coatings, films and layers. They include coating thickness (this will influence coating adhesion and fixation—the agreed optimum now seems to be within 50–100 µm), crystallinity (this affects the dissolution and biological behavior), phase and chemical purity, porosity and adhesion. The coated implants combine the surface biocompatibility and bioactivity of calcium orthophosphates with the core strength of strong substrates (Figure 12). Moreover, calcium orthophosphate coatings, films and layers decrease a release of potentially hazardous chemicals from the core implant and shield the substrate surface from environmental attack. In the case of porous implants, the coated by calcium orthophosphates surface enhances bone ingrowth into the pores [248]. The production techniques of the coatings, films and layers and their properties are now largely standardized. The clinical results for calcium orthophosphate-coated implants reveal that they have much longer life times after implantation than uncoated devices and they have been found to be particularly beneficial for younger patients. Further details on this topic might be found in special reviews [529,630,631,632,633,634].

### 5.3. Functionally Graded Bioceramics

In general, functionally gradient materials (FGMs) are defined as the materials, having gradient either compositional or structural changes from their surface to the interior of the materials. The idea of FGMs allows one device to possess two different properties. One of the most important combinations for the biomedical field is that of a mechanical strength and biocompatibility. Namely, only surface properties govern a biocompatibility of the entire device. In contrast, the strongest material determines the mechanical strength of the entire device. Although, this subject belongs to the previous section on coatings, films and layers, in a certain sense, all types of implants covered by calcium orthophosphates might be also considered as a FGM.

**Figure 13:**
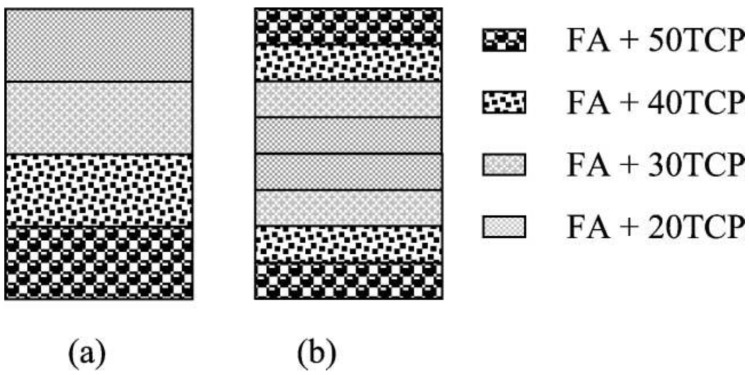
A schematic diagram showing the arrangement of the FA/β-TCP biocomposite layers. (**a**) A non-symmetric functionally gradient material (FGM); and (**b**) symmetric FGM. Reprinted from Reference [636] with permission.

Within the scope of this review, functionally graded bioceramics consisting of calcium orthophosphates solely is considered and discussed. Such formulations have been developed [92,318,502,505,575,635,636,637,638,639,640,641,642,643,644]. For example, dense sintered bodies with gradual compositional changes from α-TCP to HA were prepared by sintering a diamond-coated HA compacts at 1280 °C under a reduced pressure, followed by heating under the atmospheric conditions [635]. The content of α-TCP gradually decreased, while the content of HA increased with increasing depth from the surface. This functionally gradient bioceramics consisting of HA core and α-TCP surface showed a potential value as bone-substituting biomaterials [635]. Two types of functionally gradient FA/β-TCP biocomposites were prepared in another study [636]. As shown in Figure 13, one of the graded biocomposites was in the shape of a disk and contained four different layers of about 1 mm thick. The other graded biocomposite was also in the shape of a disk but contained two sets of the four layers, each layer being 0.5 mm thick controlled by using a certain amount of the mixed powders. The final FA/β-TCP graded structures were formed at 100 MPa and sintered at 1300 °C for 2 h [636]. Calcium orthophosphates coatings with graded crystallinity were prepared as well [642].

**Figure 14:**
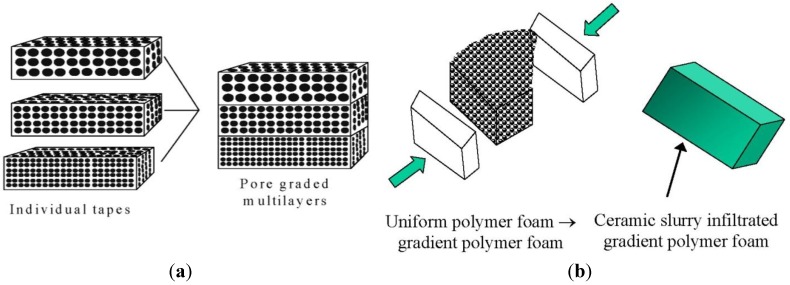
Schematic illustrations of fabrication of pore-graded bioceramics: (**a**) lamination of individual tapes, manufactured by tape casting; and (**b**) a compression molding process. Reprinted from Reference [429] with permission.

Besides, it is well known that a bone cross-section from cancellous to cortical bone is non-uniform in porosity and pore dimensions. Thus, in various attempts to mimic the porous structure of bones, calcium orthophosphate bioceramics with graded porosity have been fabricated [92,429,502,505,575,635,636,637,638,639,640,641]. For example, graded porous calcium orthophosphate bioceramics can be produced by means of tape casting and lamination (Figure 14a). Other manufacturing techniques, such as a compression molding process (Figure 14b) followed by impregnation and firing, are known as well [429]. In the first method, a HA slurry was mixed with a pore former. The mixed slurry was then cast into a tape. Using the same method, different tapes with different pore former sizes were prepared individually. The different tape layers were then laminated together. Firing was then done to remove the pore formers and sinter the HA particle compacts, resulting in graded porous bioceramics [639]. This method was also used to prepare graded porous HA with a dense part (core or layer) in order to improve the mechanical strength, as dense ceramics are much stronger than porous ceramics. However, as in the pressure infiltration of mixed particles, this multiple tape casting also has the problem of poor connectivity of pores, although the pore size and the porosity are relatively easy to control. Furthermore, the lamination step also introduces additional discontinuity of the porosity on the interfaces between the stacked layers.

Since diverse biomedical applications require different configurations and shapes, the graded (or gradient) porous bioceramics can be grouped according to both the overall shape and the structural configuration [429]. The basic shapes include rectangular blocks and cylinders (or disks). For the cylindrical shape, there are configurations of dense core—porous layer, less porous core—more porous layer, dense layer—porous core and less porous layer—more porous core. For the rectangular shape, in the gradient direction *i.e.*, the direction with varying porosity, pore size or composition, there are configurations of porous top—dense bottom (same as porous bottom—dense top), porous top—dense center—porous bottom, dense top—porous center—dense bottom, *etc.* Concerning biomedical applications, a dense core—porous layer structure is suitable for implants of a high mechanical strength and with bone ingrowth for stabilization, whereas a less porous layer—more porous core configuration can be used for drug delivery systems. Furthermore, a porous top—dense bottom structure can be shaped into implants of articulate surfaces for wear resistance and with porous ends for bone ingrowth fixation; while a dense top—porous center—dense bottom arrangement mimics the structure of head skull. Further details on bioceramics with graded porosity might be found in literature [429].

## 6. Biological Properties and *in Vivo* Behavior

The most important differences between bioactive bioceramics and all other implanted materials comprise inclusion in the metabolic processes of the organism, adaptation of either surface or the entire material to the biomedium, integration of a bioactive implant with bone tissues at the molecular level or the complete replacement of a resorbable bioceramics by healthy bone tissues. All of the enumerated processes are related to the effect of an organism on the implant. Nevertheless, another aspect of implantation is also important—the effect of the implant on the organism. For example, using of bone implants from corpses or animals, even after they have been treated in various ways, provokes a substantially negative immune reactions in the organism, which substantially limits the application of such implants. In this connection, it is useful to dwell on the biological properties of bioceramic implants, particularly those of calcium orthophosphates, which in the course of time may be resorbed completely [645].

### 6.1. Interactions with Surrounding Tissues and the Host Responses

All interactions between implants and the surrounding tissues are dynamic processes. Water, dissolved ions, various biomolecules and cells surround the implant surface within initial few seconds after the implantation. It has been accepted that no foreign material placed inside a living body is completely compatible. The only substances that conform completely are those manufactured by the body itself (autogenous), while any other substance, which is recognized as foreign, initiates some types of reactions (a host-tissue response). The reactions occurring at the biomaterial/tissue interfaces lead to time-dependent changes in the surface characteristics of both the implanted biomaterials and the surrounding tissues [76,646].

In order to develop new biomaterials, it is necessary to understand the *in vivo* host responses. Like any other species, biomaterials and bioceramics react chemically with their environment and, ideally, they should neither induce any changes nor provoke undesired reactions in the neighboring or distant tissues. In general, living organisms can treat artificial implants as biotoxic (or bioincompatible [71]), bioinert (or biostable [62]), biotolerant (or biocompatible [71]), bioactive and bioresorbable materials [1,2,3,58,59,63,66,67,68,69,70,71,645,646,647]. Biotoxic (e.g., alloys containing cadmium, vanadium, lead and other toxic elements) materials release to the body substances in toxic concentrations and/or trigger the formation of antigens that may cause immune reactions ranging from simple allergies to inflammation to septic rejection with the associated severe health consequences. They cause atrophy, pathological change or rejection of living tissue near the material as a result of chemical, galvanic or other processes. Bioinert (this term should be used with care, since it is clear that any material introduced into the physiological environment will induce a response. However, for the purposes of biomedical implants, the term can be defined as a minimal level of response from the host tissue), such as zirconia, alumina, carbon and titanium, as well as biotolerant (e.g., polymethylmethacrylate, titanium and Co-Cr alloy) materials do not release any toxic constituents but also do not show positive interaction with living tissue. They evoke a physiological response to form a fibrous capsule, thus, isolating the material from the body. In such cases, thickness of the layer of fibrous tissue separating the material from other tissues of an organism can serve as a measure of bioinertness. Generally, both bioactivity and bioresorbability phenomena are fine examples of chemical reactivity and calcium orthophosphates (both non-substituted and ion-substituted ones) fall into these two categories of bioceramics [1,2,3,58,59,63,66,67,68,69,70,71,645,646,647]. A bioactive material will dissolve slightly but promote formation of a surface layer of biological apatite before interfacing directly with the tissue at the atomic level, that result in formation of a direct chemical bonds to bones (see Section 6.4. for details). Such implants provide a good stabilization for materials that are subject to mechanical loading. A bioresorbable material will dissolve over time (regardless of the mechanism leading to the material removal) and allow a newly formed tissue to grow into any surface irregularities but may not necessarily interface directly with the material. Consequently, the functions of bioresorbable materials are to participate in dynamic processes of formation and re-absorption occurring in bone tissues; thus, bioresorbable materials are used as scaffolds or filling spacers allowing to the tissues their infiltration and substitution [67,194,324,648,649,650].

It is important to stress, that a distinction between the bioactive and bioresorbable bioceramics might be associated with structural factors only. Namely, bioceramics made from non-porous, dense and highly crystalline HA behaves as a bioinert (but a bioactive) material and is retained in an organism for at least 5–7 years without noticeable changes (Figure 2 bottom), while a highly porous bioceramics of the same composition can be resorbed approximately within a year. Furthermore, submicron-sized HA powders are biodegraded even faster than the highly porous HA scaffolds. Other examples of bioresorbable materials include porous bioceramic scaffolds made of biphasic, triphasic or multiphasic formulations (see Section 3.2) or bone grafts (dense or porous) made of CDHA [141], TCP [92,651,652] and/or ACP [481,653]. One must stress that recently the concepts of bioactive and bioresorbable materials have converged and bioactive materials are made bioresorbable, while bioresorbable materials are made bioactive [654].

Although in certain *in vivo* experiments inflammatory reactions were observed after implantation of calcium orthophosphate bioceramics [655,656,657,658,659,660,661,662], the general conclusion on using calcium orthophosphates with Ca/P ionic ratio within 1.0–1.7 is that all types of implants (bioceramics of various porosities and structures, powders or granules) are not only nontoxic but also induce neither inflammatory nor foreign-body reactions [128,663,664]. The biological response to implanted calcium orthophosphates follows a similar cascade observed in fracture healing. This cascade includes a hematoma formation, inflammation, neovascularization, osteoclastic resorption and a new bone formation. An intermediate layer of fibrous tissue between the implants and bones has been never detected. Furthermore, calcium orthophosphate implants display the ability to directly bond to bones [1,2,3,58,59,63,66,67,68,69,70,71,645,646,647]. For further details, the interested readers are referred to a good review on cellular perspectives of bioceramic scaffolds for bone tissue engineering [491].

**Figure 15:**
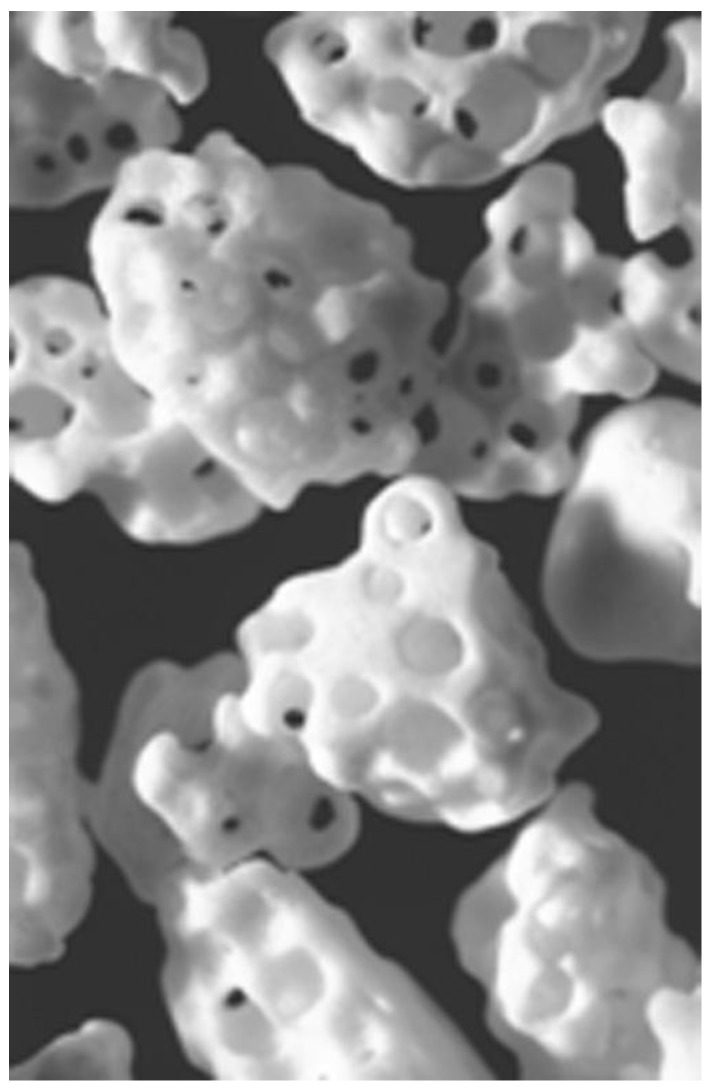
Rounded β-TCP granules of 2.6–4.8 mm in size, providing no sharp edges for combination with bone cement. Reprinted from Reference [665] with permission.

One should note that the aforementioned rare cases of the inflammatory reactions to calcium orthophosphate bioceramics were often caused by “other” reasons. For example, a high rate of wound inflammation occurred when highly porous HA was used. In that particular case, the inflammation was explained by sharp implant edges, which irritated surrounding soft tissues [656]. To avoid this, only rounded material should be used for implantation (Figure 15) [665]. Another reason for inflammation produced by porous HA could be due to micro movements of the implants, leading to simultaneous disruption of a large number of micro-vessels, which grow into the pores of the bioceramics. This would immediately produce an inflammatory reaction. Additionally, problems could arise in clinical tests connected with migration of granules used for alveolar ridge augmentation, because it might be difficult to achieve a mechanical stability of implants at the implantation sites [656]. Besides, presence of calcium pyrophosphate impurity might be the reason of inflammation [659]. Additional details on inflammatory cell responses to calcium orthophosphates might be found in a special review on this topic [660].

### 6.2. Osteoinduction

Before recently, it was generally considered, that alone, any type of synthetic bioceramics possessed neither osteogenic (osteogenesis is the process of laying down new bone material by osteoblasts) nor osteoinductive (is the property of the material to induce bone formation *de novo* or ectopically (*i.e.*, in non-bone forming sites)) properties and demonstrated a minimal immediate structural support. However, a number of reports have already shown the osteoinductive properties of certain types of calcium orthophosphate bioceramics [171,563,592,666,667,668,669,670,671,672,673,674,675,676,677,678,679,680,681,682,683,684,685] and the amount of such publications rapidly increases. For example, bone formation was found to occur in dog muscle inside porous calcium orthophosphates with surface microporosity, while bone was not observed on the surface of dense bioceramics [667]. Furthermore, implantation of porous β-TCP bioceramics appeared to induce bone formation in soft tissues of dogs, while no bone formation was detected in any α-TCP implants [668]. More to the point, titanium implants coated by a microporous layer of OCP were found to induce ectopic bone formation in goat muscles, while a smooth layer of carbonated apatite on the same implants was not able to induce bone formation there [674,675]. In another study, β-TCP powder, biphasic (HA + β-TCP) powder and intact biphasic (HA + β-TCP) rods were implanted into leg muscles of mice and dorsal muscles of rabbits [683]. One month and three months after implantation, samples were harvested for biological and histological analysis. New bone tissues were observed in 10 of 10 samples for β-TCP powder, 3 of 10 samples biphasic powder and 9 of 10 samples for intact biphasic rods at 3rd month in mice, but not in rabbits. The authors concluded that the chemical composition was the prerequisite in osteoinduction, while porosity contributed to more bone formation [683]. Therefore, researchers have already discovered the ways to prepare osteoinductive calcium orthophosphate bioceramics.

Unfortunately, the underlying mechanism(s) leading to bone induction by synthetic materials remains largely unknown. Nevertheless, besides the specific genetic factors [681] and chosen animals [683], the dissolution/precipitation behavior of calcium orthophosphates [630], their microporosity [679,683,686,687], physicochemical properties [677,679], composition [683], the specific surface area [687], nanostructure [682], as well as the surface topography and geometry [673,688,689,690] have been pointed out as the relevant parameters. A positive effect of increased microporosity on the ectopic bone formation could be both direct and indirect. Firstly, an increased microporosity is directly related to the changes in surface topography, *i.e.*, increases a surface roughness, which might affect the cellular differentiation. Secondly, an increased microporosity indirectly means a larger surface that is exposed to the body fluids leading to elevated dissolution/precipitation phenomena as compared to non-microporous surfaces. In addition, other hypotheses are also available. Namely, Reddi explained the apparent osteoinductive properties as an ability of particular bioceramics to concentrate bone growth factors, which are circulating in biological fluids, and those growth factors induce bone formation [688]. Other researchers proposed a similar hypothesis that the intrinsic osteoinduction by calcium orthophosphate bioceramics is a result of adsorption of osteoinductive substances on their surface [673]. Moreover, Ripamonti [689] and Kuboki *et al.* [690] independently postulated that the geometry of calcium orthophosphate bioceramics is a critical parameter in bone induction. Specifically, bone induction by calcium orthophosphates was never observed on flat bioceramic surfaces. All osteoinductive cases were observed on either porous structures or structures contained well-defined concavities. What’s more, bone formation was never observed on the peripheries of porous implants and was always found inside the pores or concavities, aligning the surface [194]. Some researchers speculated that a low oxygen tension in the central region of implants might provoke a dedifferentiation of pericytes from blood micro-vessels into osteoblasts [691]. Finally but yet importantly, both nano-structured rough surfaces and a surface charge on implants were found to cause an asymmetrical division of the stem cells into osteoblasts, which is important for osteoinduction [686].

Nevertheless, to finalize this topic, it is worth citing a conclusion made by Boyan and Schwartz [692] (p. 9): “Synthetic materials are presently used routinely as osteoconductive bone graft substitutes, but before purely synthetic materials can be used to treat bone defects in humans where an osteoinductive agent is required, a more complete appreciation of the biology of bone regeneration is needed. An understanding is needed of how synthetic materials modulate the migration, attachment, proliferation and differentiation of mesenchymal stem cells, how cells on the surface of a material affect other progenitor cells in the peri-implant tissue, how vascular progenitors can be recruited and a neovasculature maintained, and how remodeling of newly formed bone can be controlled.”

### 6.3. Biodegradation

Shortly after implantation, a healing process is initiated by compositional changes of the surrounding bio-fluids and adsorption of biomolecules. Following this, various types of cells reach the bioceramic surface and the adsorbed layer dictates the ways the cells respond. Further, a biodegradation of the implanted bioceramics begins. This process can occur by either physicochemical dissolution with a possibility of phase transformations or cellular activity (so called, bioresorption). More likely, a combination of both processes takes place *in vivo*. Since the existing calcium orthophosphates are differentiated by Ca/P ratio, basicity/acidity and solubility (Table 1), their degradation kinetics and mechanism depend on the chosen type of calcium orthophosphate [693,694]. Since dissolution is a physical chemistry process, it is controlled by some factors, such as solubility, surface area to volume ratio, local acidity, fluid convection and temperature. For HA and FA, the dissolution mechanism in acids has been described by a sequence of four successive chemical equations, in which several other calcium orthophosphates, such as TCP, DCPD/DCPA and MCPM/MCPA, appear as virtual intermediate phases [695,696].

With a few exceptions, dissolution rates of calcium orthophosphates are inversely proportional to the Ca/P ratio (except of TTCP), phase purity and crystalline size, as well as it is directly related to both the porosity and the surface area. In addition, phase transformations might occur with OCP, DCPA, DCPD, α-TCP, β-TCP and ACP because they are unstable in aqueous environment under the physiological conditions. Bioresorption is a biological process mediated by cells (mainly, osteoclasts and, in a lesser extent, macrophages) [697,698]. It depends on the response of cells to their environment. Osteoclasts attach firmly to the implant and dissolve calcium orthophosphates by secreting an enzyme carbonic anhydrase or any other acid, leading to a local pH drop to ~4–5 [699]. Furthermore, nanodimensional particles of calcium orthophosphate can also be phagocytosed by cells, *i.e.*, they are incorporated into cytoplasm and thereafter dissolved by acid attack and/or enzymatic processes [700]. In any case, *in vivo* biodegradation of calcium orthophosphates is a complicated combination of various non-equilibrium processes, occurring simultaneously and/or in competition with each other.

Usually, an *in vitro* biodegradation of calcium orthophosphate bioceramics is simulated by suspending the material in a slightly acidic (pH ~4) buffer and monitoring the release of major ions with time [181,694,701,702,703]. The acidic buffer, to some extent, mimics the acidic environment during osteoclastic activity. In one study, an *in vivo* behavior of porous β-TCP bioceramics prepared from rod-shaped particles and that prepared from non-rod-shaped particles in the rabbit femur was compared. Although the porosities of both types of β-TCP bioceramics were almost the same, a more active osteogenesis was preserved in the region where rod-shaped bioceramics was implanted [704]. This result implied that the microstructure affected the activity of bone cells and subsequent bone replacement.

The experimental results demonstrated that both the dissolution kinetics and *in vivo* biodegradation of biologically relevant calcium orthophosphates proceed in the following decreasing order: β-TCP > bovine bone apatite (unsintered) > bovine bone apatite (sintered) > coralline HA > HA. In the case of biphasic (HA + TCP), triphasic and multiphasic calcium orthophosphates, the biodegradation kinetics depends on the HA/TCP ratio: the higher the ratio, the lower the degradation rate. Similarly, *in vivo* degradation rate of biphasic TCP (α-TCP + β-TCP) bioceramics appeared to be lower than that of α-TCP and higher than that of β-TCP bioceramics, respectively [112]. Furthermore, incorporation of doping ions can either increase (e.g., CO_3_^2−^, Mg^2+^ or Sr^2+^) or decrease (e.g., F^−^) the solubility (therefore, biodegradability) of CDHA and HA. Contrarily to apatites, solubility of β-TCP is decreased by incorporation of either Mg^2+^ or Zn^2+^ ions [563]. Here, one should remind that ion-substituted calcium orthophosphates are not considered in this review; the interested readers are advised to read the original publications [26,27,28,29,30,31,32,33,34,35,36,37,38,39,40,41,42,43,44,45,46,47,48,49,50,51,52,53,54,55,56,57].

### 6.4. Bioactivity

Generally, bioactive materials interact with surrounding bone resulting in formation of a chemical bond to this tissue (bone bonding). The bioactivity phenomenon is determined by both chemical factors, such as crystal phases and molecular structures of a biomaterial, and physical factors, such as surface roughness and porosity. Currently, it is agreed that the newly formed bone bonds directly to biomaterials through a carbonated CDHA layer precipitating at the bone/biomaterial interface. Strange enough but a careful seeking in the literature resulted in just a few publications [563,705,706,707], where the bioactivity mechanism of calcium orthophosphates was briefly described. For example, the chemical changes occurring after exposure of a synthetic HA bioceramics to both *in vivo* (implantation in human) and *in vitro* (cell culture) conditions were studied. A small amount of HA was phagocytozed but the major remaining part behaved as a secondary nucleator as evidenced by the appearance of a newly formed mineral [705]. *In vivo*, a cellular activity (e.g., of macrophages or osteoclasts) associated with an acidic environment were found to result in partial dissolution of calcium orthophosphates, causing liberation of calcium and orthophosphate ions to the microenvironment. The liberated ions increased a local supersaturation degree of the surrounding biologic fluids, causing precipitation of nano-sized crystals of biological apatite with simultaneous incorporating of various ions presented in the fluids. Infrared spectroscopic analyses demonstrated that these nanodimensional crystals were intimately associated with bioorganic components (probably proteins), which might also have originated from the biologic fluids, such as serum [563].

**Figure 16:**
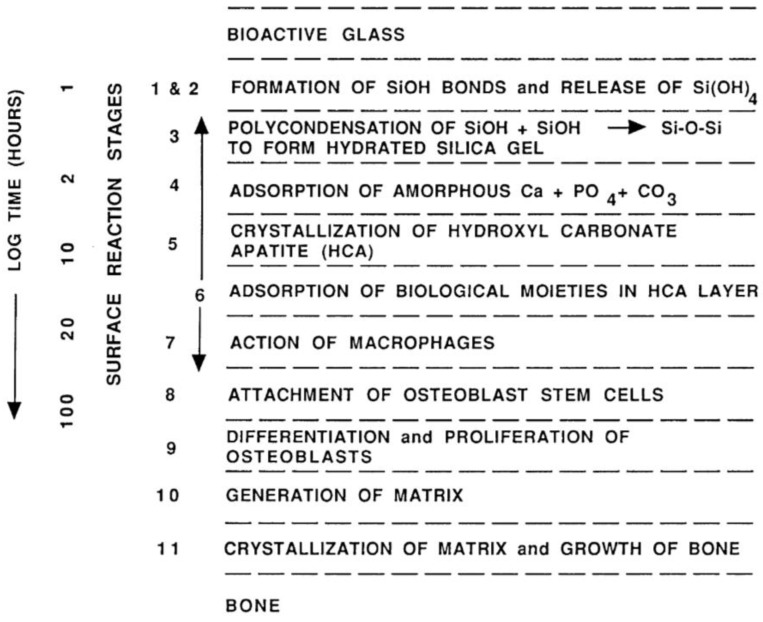
A sequence of interfacial reactions involved in forming a bond between tissue and bioactive ceramics. Reprinted from References [66,67,68,69] with permission.

Therefore, one should consider the bioactivity mechanism of other biomaterials, particularly of bioactive glasses—the concept introduced by Prof. Larry L. Hench [66,67,68,69]. The bonding mechanism of bioactive glasses to living tissues involves a sequence of 11 successive reaction steps (Figure 16), some of which comprise calcium orthophosphates. The initial five steps occurred on the surface of bioactive glasses are “chemistry” only, while the remaining six steps belong to “biology” because the latter include colonization by osteoblasts, followed by proliferation and differentiation of the cells to form a new bone that had a mechanically strong bond to the implant surface. Therefore, in the case of bioactive glasses the border between “dead” and “alive” is postulated between stages 5 and 6. According to Hench, all bioactive materials “form a bone-like apatite layer on their surfaces in the living body and bond to bone through this apatite layer. The formation of bone-like apatite on artificial material is induced by functional groups, such as Si–OH (in the case of biological glasses), Ti–OH, Zr–OH, Nb–OH, Ta–OH, –COOH and –H_2_PO_4_ (in the case of other materials). These groups have specific structures revealing negatively charge and induce apatite formation via formations of an amorphous calcium compound, e.g., calcium silicate, calcium titanate and ACP” [66,67,68,69].

**Figure 17:**
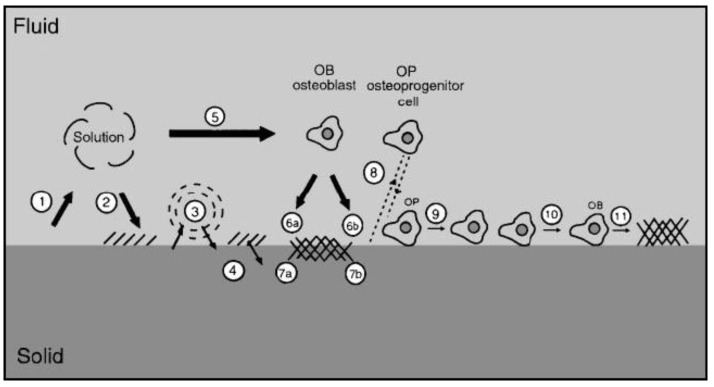
A schematic diagram representing the events, which take place at the interface between bioceramics and the surrounding biological environment: (**1**) dissolution of bioceramics; (**2**) precipitation from solution onto bioceramics; (**3**) ion exchange and structural rearrangement at the bioceramic/tissue interface; (**4**) interdiffusion from the surface boundary layer into the bioceramics; (**5**) solution-mediated effects on cellular activity; (**6**) deposition of either the mineral phase (**a**) or the organic phase (**b**) without integration into the bioceramic surface; (**7**) deposition with integration into the bioceramics; (**8**) chemotaxis to the bioceramic surface; (**9**) cell attachment and proliferation; (**10**) cell differentiation; and (**11**) extracellular matrix formation. All phenomena, collectively, lead to the gradual incorporation of a bioceramic implant into developing bone tissue. Reprinted from Reference [76] with permission.

**Figure 18:**
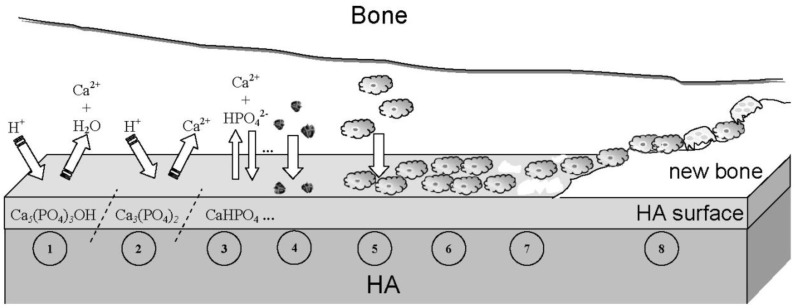
A schematic diagram representing the phenomena that occur on HA surface after implantation: (**1**) beginning of the implant procedure, where a solubilization of the HA surface starts; (**2**) continuation of the solubilization of the HA surface; (**3**) the equilibrium between the physiological solutions and the modified surface of HA has been achieved (changes in the surface composition of HA does not mean that a new phase of DCPA or DCPD forms on the surface); (**4**) adsorption of proteins and/or other bioorganic compounds; (**5**) cell adhesion; (**6**) cell proliferation; (**7**) beginning of a new bone formation; and (**8**) new bone has been formed. Reprinted from Reference [707] with permission.

In addition, one should mention another set of 11 successive reaction steps for bonding mechanism of unspecified bioceramics, developed by Prof. Paul Ducheyne (Figure 17) [76]. One can see that the Ducheyne’s model is rather similar to that proposed by Hench; however, there are noticeable differences between them. For example, Ducheyne mentions on ion exchange and structural rearrangement at the bioceramic/tissue interface (stage 3), as well as on interdiffusion from the surface boundary layer into bioceramics (stage 4) and deposition with integration into the bioceramics (stage 7), which are absent in the Hench’s model. On the other hand, Hench describes six biological stages (stages 6–11), while Ducheyne describes only four ones (stages 8–11). Both models have been developed almost 2 decades ago and, to the best of my knowledge, remain unchanged since then. Presumably, both approaches have *pro et contra* of their own and, obviously, should be updated and/or revised. Furthermore, in literature there are at least two other descriptions of the biological and cellular events occurring at the bone/implant interface [708,709]. Unfortunately, both of them comprise lesser number of stages. In 2010, one more hypothesis has been proposed (Figure 18). For the first time, it describes reasonable surface transformations, happening with calcium orthophosphate bioceramics (in that case, HA) shortly after the implantation [707].

An important study on formation of calcium orthophosphate precipitates on various types of bioceramic surfaces in both simulated body fluid (SBF) and rabbit muscle sites was performed [710]. The bioceramics were sintered porous solids, including bioglass, glass-ceramics, α-TCP, β-TCP and HA. An ability to induce calcium orthophosphate precipitation was compared among these types of bioceramics. The following conclusions were made: (1) OCP formation ubiquitously occurred on all types of bioceramic surfaces both *in vitro* and *in vivo*, except on β-TCP; (2) Apatite formation did not occur on every type of bioceramic surface; it was less likely to occur on the surfaces of HA and α-TCP; (3) Precipitation of calcium orthophosphates on the bioceramic surfaces was more difficult *in vivo* than *in vitro*; (4) Differences in calcium orthophosphate precipitation among the bioceramic surfaces were less noticeable *in vitro* than that *in vivo*; and (5) β-TCP bioceramics showed a poor ability of calcium orthophosphate precipitation both *in vitro* and *in vivo* [710]. These findings clearly revealed that apatite formation in the physiological environments could not be confirmed as the common feature of bioceramics. Nevertheless, for want of anything better, currently the bioactivity mechanism of calcium orthophosphate bioceramics should be described by a reasonable combination of Figure 16, Figure 17 and Figure 18, e.g., by updating the Ducheyne’s and Hench’s models by three initial stages taken from Figure 18.

Interestingly that the bioactivity of HA bioceramics might be enhanced by a high-energy ion irradiation [711]. The effect was attributed to formation of a unique 3D macroporous apatite layer of decreased crystallinity and crystal size on the irradiated surfaces. Obviously, to get further insights into the bioactivity phenomenon, the atomic and molecular processes occurring at the bioceramic surface in aqueous solutions and their effects on the relevant reaction pathways of cells and tissues must be elucidated in more details.

### 6.5. Cellular Response

Fixation of any implants in the body is a complex dynamic process that remodels the interface between the implants and living tissues at all dimensional levels, from the molecular up to the cell and tissue morphology level, and at all time scales, from the first second up to several years after implantation. Immediately following the implantation, a space filled with biological fluids appears next to the implant surface. With time, cells are adsorbed at the implant surface that will give rise to their proliferation and differentiation towards bone cells, followed by revascularisation and eventual gap closing. Ideally, a strong bond is formed between the implants and surrounding tissues [71]. An interesting study on the interfacial interactions between calcined HA and substrates has been performed [712], where the interested readers are referred for further details.

The aforementioned paragraph clearly demonstrates an importance of studies on cellular responses to calcium orthophosphate bioceramics. Therefore, such investigations have been performed extensively for several decades [660,713,714,715,716,717,718,719,720,721,722,723,724]. For example, bioceramic discs made of 7 different calcium orthophosphates (TTCP, HA, carbonate apatite, β-TCP, α-TCP, OCP and DCPD) were incubated in osteoclastic cell cultures for 2 days. In all cases, similar cell morphologies and good cell viability were observed; hoverer, different levels of resorbability of various calcium orthophosphates were detected [716]. Similar results were found for fluoridated HA coatings [718]. Experiments performed with human osteoblasts revealed that nanostructured bioceramics prepared from nano-sized HA showed significant enhancement in mineralization compared to microstructured HA bioceramics [717]. In addition, the influence of lengths and surface areas of rod-shaped HA on cellular response was studied. Again, similar cell morphologies and good cell viability were observed; however, it was concluded that high surface area could increase cell-particle interaction [721]. Nevertheless, another study with cellular response to rod-shaped HA bioceramics, revealed that some types of crystals might trigger a severe inflammatory response [722]. In addition, calcium orthophosphate-based sealers appeared to show less cytotoxicity and inflammatory mediators compared with other sealers [719]. More examples are available in literature.

Cellular biodegradation of calcium orthophosphate bioceramics is known to depend on its phases. For example, a higher solubility of β-TCP was shown to prevent L-929 fibroblast cell adhesion, thereby leading to damage and rupture of the cells [725]. A mouse ectopic model study indicated the maximal bone growth for the 80:20 β-TCP:HA biphasic formulations preloaded with human mesenchymal stem cells when compared to other calcium orthophosphates [726]. The effects of substrate microstructure and crystallinity have been corroborated with an *in vivo* rabbit femur model, where rod-like crystalline β-TCP was reported to enhance osteogenesis when compared to non-rod like crystalline β-TCP [704]. Additionally, using a dog mandibular defect model, a higher bone formation on a scaffold surface coated by nanodimensional HA was observed when compared to that coated by a micro-dimensional HA [727]. Furthermore, studies revealed a stronger stress signaling response by osteoblast precursor cells in 3D scaffolds when compared to 2D surfaces [728].

Mesenchymal stem cells are one of the most attractive cellular lines for application as bone grafts [729,730]. Early investigations by Okumura *et al.*, indicated an adhesion, proliferation and differentiation, which ultimately became new bone and integrated with porous HA bioceramics [714]. Later, a sustained co-culture of endothelial cells and osteoblasts on HA scaffolds for up to 6 weeks was demonstrated [731]. Furthermore, a release of factors by endothelial and osteoblast cells in co-culture supported proliferation and differentiation was suggested to ultimately result in microcapillary-like vessel formation and supported a neo-tissue growth within the scaffold [491]. More to the point, investigation of rat calvaria osteoblasts cultured on transparent HA bioceramics, as well as the analysis of osteogenic-induced human bone marrow stromal cells at different time points of culturing indicated to a good cytocompatibility of HA bioceramics and revealed favorable cell proliferation [421]. The positive results for other types of cells have been obtained in other studies [203,416,417,420,452,453,454,732,733,734].

Interestingly that HA scaffolds with marrow stromal cells in a perfused environment were reported to result in ~85% increase in mean core strength, a ~130% increase in failure energy and a ~355% increase in post-failure strength. The increase in mineral quantity and promotion of the uniform mineral distribution in that study was suggested to attribute to the perfusion effect [586]. Additionally, other investigators indicated to mechanical properties increasing for other calcium orthophosphate scaffolds after induced osteogenesis [585,588].

To finalize this section, one should mention on the newest developments to influence the cellular response. First, to facilitate interactions with cells, the calcium orthophosphate surface might be functionalized [735,736,737,738]. Second, it appears that crystals of biological apatite of calcified tissues exhibit different orientations depending on the tissue. Namely, in vertebrate bones and tooth enamel surfaces, the respective *a,b*-planes and *c*-planes of the apatite crystals are preferentially exposed. Therefore, ideally, this should be taken into account in artificial bone grafts. Recently, a novel process to fabricate dense HA bioceramics with highly preferred orientation to the *a,b*-plane was developed [739,740]. The results revealed that increasing the *a,b*-plane orientation degree shifted the surface charge from negative to positive and decreased the surface wettability with simultaneous decreasing of cell attachment efficiency [740].

## 7. Non-Biomedical Applications

Due to their strong adsorption ability, surface acidity or basicity and ion exchange abilities, both hydroxyapatite and other calcium orthophosphates appear to possess a catalytic activity [25,741,742,743,744,745,746,747,748,749,750,751,752]. As seen from the references, calcium orthophosphates are able to catalyze oxidation and reduction reactions, as well as formation of C–C bonds. Namely, the application in oxidation reactions mainly includes oxidation of alcohol and dehydrogenation of hydrocarbons, while the reduction reactions include hydrogenolysis and hydrogenation. The formation of C–C bonds mainly comprises Claisen-Schmidt and Knoevenagel condensation reactions, Michael addition reaction, as well as Friedel-Crafts, Heck, Diels-Alder and adol reactions [749].

In addition, due to the chemical similarity to the inorganic part of mammalian calcified tissues, both hydroxyapatite and other calcium orthophosphates appear to be good solid carriers for chromatography of biological substances. Namely, such high-value biological materials, as recombinant proteins, therapeutic antibodies and nucleic acids are separated and purified [753,754,755,756,757,758]. However, since both subjects are almost irrelevant to bioceramics, they are not detailed further.

## 8. Calcium Orthophosphate Bioceramics in Tissue Engineering

### 8.1. Tissue Engineering

Tissue/organ repair has been the ultimate goal of surgery from ancient times to nowadays [74,75]. The repair has traditionally taken two major forms: tissue grafting followed by organ transplantation and alloplastic or synthetic material replacement. Both approaches, however, have limitations. Grafting requires second surgical sites with associated morbidity and is restricted by limited amounts of material, especially for organ replacement. Synthetic materials often integrate poorly with host tissue and fail over time due to wear and fatigue or adverse body response [759]. In addition, all modern orthopedic implants lack three of the most critical abilities of living tissues: (i) self-repairing; (ii) maintaining of blood supply; and (iii) self-modifying their structure and properties in response to external aspects such as a mechanical load [760]. Needless to mention, that bones not only possess all of these properties but, in addition, they are self-generating, hierarchical, multifunctional, nonlinear, composite and biodegradable; therefore, the ideal artificial bone grafts must possess similar properties [79].

The last decades have seen a surge in creative ideas and technologies developed to tackle the problem of repairing or replacing diseased and damaged tissues, leading to the emergence of a new field in healthcare technology now referred to as *tissue engineering*. This is an interdisciplinary field that exploits a combination of living cells, engineering materials and suitable biochemical factors in a variety of ways to improve, replace, restore, maintain or enhance living tissues and whole organs [761,762,763]. However, as two of three major components (namely, cells and biochemical factors) of the tissue engineering subject appear to be far beyond the scope of this review, the topic of tissue engineering is narrowed down to the engineering materials prepared from calcium orthophosphate bioceramics only.

Regeneration, rather than a repair, is the central goal of any tissue engineering strategy. Thus, tissue engineering has a potential to create tissues and organs *de novo* [762]. This field of science started more than two decades ago [764,765] and the famous publication by Langer and Vacanti [766] has greatly contributed to the promotion of tissue engineering research worldwide. The field of tissue engineering, particularly when applied to bone substitutes where tissues often function in a mechanically demanding environment [767,768,769], requires a collaboration of excellence in cell and molecular biology, biochemistry, material sciences, bioengineering and clinical research. For the success, it is necessary that researchers with expertise in one area have an appreciation of the knowledge and challenges of the other areas. However, since the technical, regulatory and commercial challenges might be substantial, the introduction of new products is likely to be slow [762].

Nowadays, tissue engineering is at full research potential due to the following key advantages: (i) the solutions it provides are long-term, much safer than other options and cost-effective as well; (ii) the need for a donor tissue is minimal, which eliminates the immuno-suppression problems; and(iii) the presence of residual foreign material is eliminated as well [770,771].

### 8.2. Scaffolds and Their Properties

It would be very convenient to both patients and physicians if devastated tissues or organs of patients can be regenerated by simple cell injections to the target sites but such cases are rare. The majority of large-sized tissues and organs with distinct 3D form require a support for their formation from cells. The support is called scaffold, template and/or artificial extracellular matrix [148,149,553,764,767,768,769,770,771,772,773,774,775]. The major function of scaffolds is similar to that of the natural extracellular matrix that assists proliferation, differentiation and biosynthesis of cells. In addition, scaffolds placed at the regeneration sites will prevent disturbing cells from invasion into the sites of action [776,777]. The role of scaffolds has been perfectly described by Andrés Segovia (1893–1987), a Spanish classical guitarist: “When one puts up a building one makes an elaborate scaffold to get everything into its proper place. But when one takes the scaffold down, the building must stand by itself with no trace of the means by which it was erected. That is how a musician should work.”

Therefore, the idea behind tissue engineering is to create or engineer autografts by either expanding autologous cells *in vitro* guided by a scaffold or implanting an acellular template *in vivo* and allowing the patient’s cells to repair the tissue guided by the scaffold. The first phase is the *in vitro* formation of a tissue construct by placing the chosen cells and scaffolds in a metabolically and mechanically supportive environment with growth media (in a bioreactor), in which the cells proliferate and elaborate extracellular matrix. It is expected that cells infiltrate into the porous matrix and consequently proliferate and differentiate therein [778,779]. In the second phase, the construct is implanted in the appropriate anatomic location, where remodeling *in vivo* is intended to recapitulate the normal functional architecture of an organ or a tissue [780,781]. The key processes occurring during both *in vitro* and *in vivo* phases of the tissue formation and maturation are: (1) cell proliferation, sorting and differentiation; (2) extracellular matrix production and organization; (3) biodegradation of the scaffold; and (4) remodeling and potentially growth of the tissue [782].

To achieve the goal of tissue reconstruction, the scaffolds must meet several specific requirements [148,149,772]. A reasonable surface roughness is necessary to facilitate cell seeding and fixation [783,784,785,786,787]. A sufficient mechanical strength and stiffness are mandatory to oppose contraction forces and later for the remodeling of damaged tissues [788,789]. A high porosity and an adequate pore dimensions (Table 2 and Table 4) are very important to allow cell migration, vascularization, as well as a diffusion of nutrients [440]. A French architect Robert le Ricolais (1894–1977) stated: “The art of structure is where to put the holes”. Therefore, to enable proper tissue ingrowth, vascularization and nutrient delivery, scaffolds should have a highly interconnected porous network, formed by a combination of macro- and micropores, in which more than ~60% of the pores should have a size ranging from ~150 μm to ~400 μm and at least ~20% should be smaller than ~20 μm [28,440,451,452,456,549,550,551,555,557,563,589,590,591,592,593,594,595,669,759,790,791,792,793,794,795,796,797,798,799,800]. In addition, scaffolds must be manufactured from the materials with controlled biodegradability and/or bioresorbability, such as calcium orthophosphates, so that a new bone will eventually replace the scaffold [767,794,801]. Furthermore, the degradation by-products of scaffolds must be non-cytotoxic. More to the point, the resorption rate has to coincide as much as possible with the rate of bone formation (*i.e.*, between a few months and about 2 years) [802]. This means that while cells are fabricating their own natural matrix structure around themselves, the scaffold is able to provide a structural integrity within the body and eventually it will break down leaving the newly formed tissue that will take over the mechanical load. However, one should bear in mind that the scaffold’s architecture changes with the degradation process and the degradation by-products affect the biological response. Besides, scaffolds should be easily fabricated into a variety of shapes and sizes [803] and be malleable to fit irregularly shaped defects. In many cases, ease of processability, as well as easiness of conformation and injectability, such as self-setting calcium orthophosphate formulations possess (see Section 5.1), can determine the choice of a certain biomaterial. Finally, sterilization with no loss of properties is a crucial step in scaffold production at both a laboratory and an industrial level [767,768,769]. Thus, each scaffold should fulfill many functions before, during and after implantation.

**Table 4:** A hierarchical pore size distribution that an ideal scaffold should exhibit [28].

Number	Pore sizes of a 3D scaffold	A biochemical effect or function
1	<1 μm	Interaction with proteins
		Responsible for bioactivity
2	1–20 μm	Type of cells attracted
		Cellular development
		Orientation and directionality of cellular ingrowth
3	100–1,000 μm	Cellular growth
		Bone ingrowth
		Predominant function in the mechanical strength
4	>1,000 μm	Implant functionality
		Implant shape
		Implant esthetics

**Figure 19:**
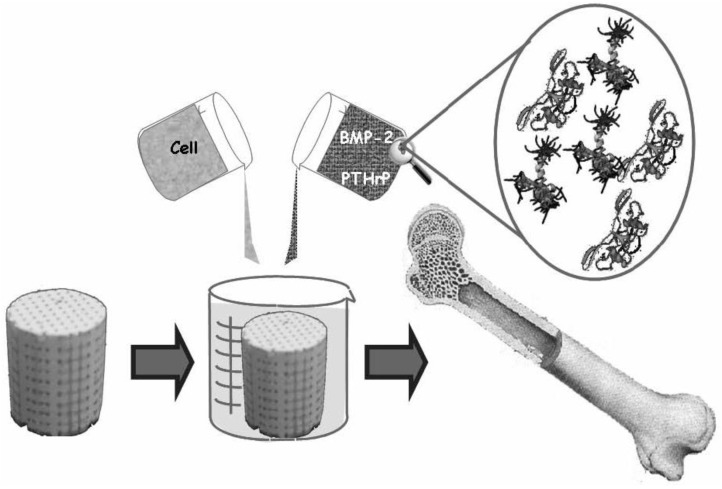
A schematic view of a third generation biomaterial, in which porous calcium orthophosphate bioceramics acts as a scaffold or a template for cells, growth factors, *etc.* Reprinted from Reference [62] with permission.

Many fabrication techniques are available to produce porous calcium orthophosphate scaffolds (Table 2) with varying architectural features (for details, see Section 3.3). In order to achieve the desired properties at minimum expenses, the production process should be optimized [804]. With the advent of tissue engineering, the search is on for the ultimate option—a “tissue engineered bone substitute”, consisting of a synthetic calcium orthophosphate scaffold impregnated with cells and growth factors. Figure 19 schematically depicts a possible fabrication process of such item that, afterwards, will be implanted into a living organism to induce bone regeneration [62].

**Figure 20:**
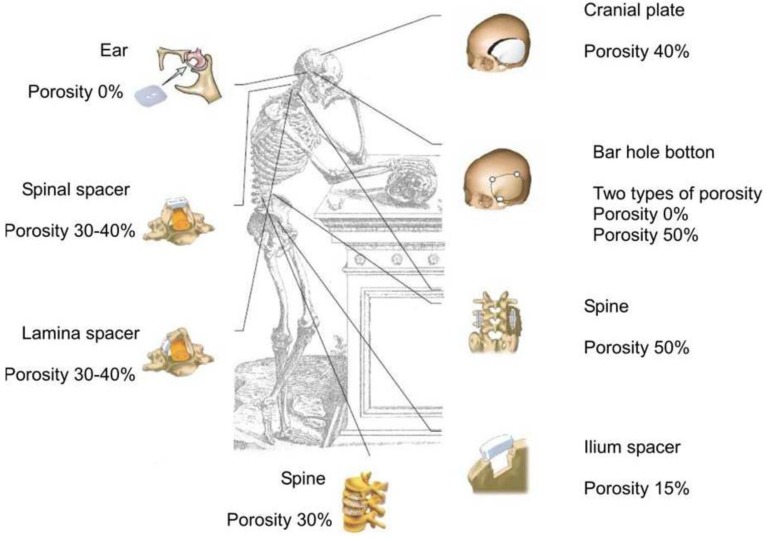
A schematic drawing presenting the potential usage of HA with various degrees of porosity. Reprinted from Reference [542] with permission.

To finalize this topic, one should mention on fundamental unfeasibility to create so-called “ideal scaffold” for bone grafting. For instance, bones of human skeleton have very different dimensions, shapes and structures depending on their functions and locations. Therefore, synthetic bone grafts of various sizes, shapes, porosity, mechanical strength, composition and resorbability appear to be necessary. Thus, HA bioceramics of 0% to 15% porosity is used as both ilium and intervertebral spacers, where a high strength is required, HA bioceramics of 30% to 40% porosity is useful as spinous process spacer for laminoplasty, where both bone formation and middle strength are necessary, while HA bioceramics of 40% to 60% porosity is useful for the calvarias plate, where a fast bone formation is needed (Figure 20) [542]. Furthermore, defining the optimum parameters for artificial scaffolds is in fact an attempt to find a reasonable compromise between various conflicting functional requirements. Namely, an increased mechanical strength of bone substitutes requires solid and dense structures, while colonization of their surfaces by cells requires interconnected porosity. Additional details and arguments on this subject are well described in a recent publication [805] (p. 478), in which the authors concluded: “there is enough evidence to postulate that ideal scaffold architecture does not exist.”

### 8.3. Bioceramic Scaffolds from Calcium Orthophosphates

Philosophically, the increase in life expectancy requires biological solutions to all biomedical problems, including orthopedic ones, which were previously managed with mechanical solutions. Therefore, since the end of 1990s, the biomaterials research focuses on tissue regeneration instead of tissue replacement [806]. The alternatives include use hierarchical bioactive scaffolds to engineer *in vitro* living cellular constructs for transplantation or use bioresorbable bioactive particulates or porous networks to activate *in vivo* the mechanisms of tissue regeneration [807,808]. Thus, the aim of calcium orthophosphates is to prepare artificial porous bioceramic scaffolds able to provide the physical and chemical cues to guide cell seeding, differentiation and assembly into 3D tissues of a newly formed bone [463,727,809,810,811,812,813,814,815,816,817]. Particle sizes, shape and surface roughness of the scaffolds are known to affect cellular adhesion, proliferation and phenotype [783,784,785,786,787]. Additionally, the surface energy might play a role in attracting particular proteins to the bioceramic surface and, in turn, this will affect the cells affinity to the material. More to the point, cells are exceedingly sensitive to the chemical composition and their bone-forming functions can be dependent on grain morphology of the scaffolds. For example, osteoblast functions were found to increase on nanodimensional fibers if compared to nanodimensional spheres because the former more closely approximated the shape of biological apatite in bones [818]. Besides, a significantly higher osteoblast proliferation on HA bioceramics sintered at 1200 °C as compared to that on HA bioceramics sintered at 800 °C and 1000 °C was reported [819]. Furthermore, since ions of calcium and orthophosphate are known to regulate bone metabolism, calcium orthophosphates appear to be among the few bone graft substitute materials, which can be considered as a drug.

Thus, to meet the tissue engineering requirements, much attention is devoted to further improvements of calcium orthophosphate bioceramics [820,821]. From the chemical point of view, the developments include synthesis of novel ion-substituted calcium orthophosphates [26,27,28,29,30,31,32,33,34,35,36,37,38,39,40,41,42,43,44,45,46,47,48,49,50,51,52,53,54,55,56,57]. From the material point of view, the major research topics include nanodimensional and nanocrystalline structures [822,823,824], amorphous compounds [825], organic-inorganic biocomposites and hybrid biomaterials [353], biphasic, triphasic and multiphasic formulations [121], as well as various types of structures, forms and shapes. The latter comprise fibers, whiskers and filaments [241,826,827,828,829,830,831,832,833,834,835,836,837,838,839], macro-, micro- and nano-sized spheres, beads and granules [839,840,841,842,843,844,845,846,847,848,849,850,851,852,853,854,855,856], micro- and nano-sized tubes [857,858,859,860], porous 3D scaffolds made of ACP [481,653,861], TCP [86,89,161,528,529,862], HA [167,455,456,498,530,531,532,804,863,864,865,866,867,868,869] and biphasic formulations [251,489,502,552,814,850,870,871,872,873,874], structures with graded porosity [92,429,502,505,575,635,636,637,638,639,640,641] and hierarchically organized ones [875,876]. Furthermore, an addition of defects through an intensive milling [877,878] or their removal by a thermal treatment [879] can be used to modify a chemical reactivity of calcium orthophosphates. Besides, more attention should be paid to a crystallographically aligned calcium orthophosphate bioceramics [880].

There are three principal therapeutic strategies for treating diseased or injured tissues in patients: (i) implantation of freshly isolated or cultured cells; (ii) implantation of tissues assembled *in vitro* from cells and scaffolds; and (iii) *in situ* tissue regeneration. For cellular implantation, individual cells or small cellular aggregates from the patient or a donor are either injected into the damaged tissue directly or are combined with a degradable scaffold *in vitro* and then implanted. For tissue implantation, a complete 3D tissue is grown *in vitro* using patient or donor cells and a bioresorbable scaffold and then is implanted into the patients to replace diseased or damaged tissues. For *in situ* regeneration, a scaffold implanted directly into the injured tissue stimulates the body’s own cells to promote local tissue repair [327,761]. In any case, simply trapping cells at the particular point on a surface is not enough: the cells must be encouraged to differentiate, which is impossible without the presence of suitable biochemical factors [881]. All previously mentioned clearly indicates that for the purposes of tissue engineering, calcium orthophosphate bioceramics plays an auxiliary role; namely, it acts as a suitable material to manufacture the appropriate 3D templates, substrates or scaffolds to be colonized by living cells before the successive implantation [882,883,884]. The *in vitro* evaluation of potential calcium orthophosphate scaffolds for tissue engineering has been described elsewhere [885], while the data on the mechanical properties of calcium orthophosphate bioceramics for use in tissue engineering are also available [815,886,887]. The effect of a HA-based biomaterial on gene expression in osteoblast-like cells was reported as well [888]. To conclude this part, the excellent biocompatibility of calcium orthophosphate bioceramics, its possible osteoinductivity [171,563,592,666,667,668,669,670,671,672,673,674,675,676,677,678,679,680,681,682,683,684,685] and a high affinity for drugs [72,73,74,75,889,890], proteins and cells [891] make them very functional for the tissue engineering applications. The feasible production of scaffolds with tailored structures and properties opens up a spectacular future for calcium orthophosphates [888,889,890,891,892,893,894,895,896,897].

### 8.4. A Clinical Experience

To date, there are just a few publications on clinical application of cell-seeded calcium orthophosphate bioceramics for bone tissue engineering of humans. Namely, Quarto *et al.* [898] were the first to report a treatment of large (4–7 cm) bone defects of the tibia, ulna and humerus in three patients from 16 to 41 years old, where the conventional surgical therapies had failed. The authors implanted a custom-made unresorbable porous HA scaffolds seeded with *in vitro* expanded autologous bone marrow stromal cells. In all three patients, radiographs and computed tomographic scans revealed abundant callus formation along the implants and good integration at the interfaces with the host bones by the second month after surgery [898]. In the same year, Vacanti *et al.* [899] reported the case of a man who had a traumatic avulsion of the distal phalanx of a thumb. The phalanx was replaced with a specially treated natural coral (porous HA; 500-pore ProOsteon (see Table 3)) implant that was previously seeded with *in vitro* expanded autologous periosteal cells. The procedure resulted in the functional restoration of a stable and biomechanically sound thumb of normal length, without the pain and complications that are usually associated with harvesting a bone graft.

Morishita *et al.* [900] treated a defect resulting from surgery of benign bone tumors in three patients using HA scaffolds seeded with *in vitro* expanded autologous bone marrow stromal cells after osteogenic differentiation of the cells. Two bone defects in a tibia and one defect in a femur were treated. Although ectopic implants in nude mice were mentioned to show the osteogenicity of the cells, details such as the percentage of the implants containing bone and at what quantities were not reported. Furthermore, cell-seeded calcium orthophosphate scaffolds were found to be superior to autograft, allograft or cell-seeded allograft in terms of bone formation at ectopic implantation sites [901]. Besides, it has been hypothesized that dental follicle cells combined with β-TCP bioceramics might become a novel therapeutic strategy to restore periodontal defects [902]. In still another study, the behavior of human periodontal ligament stem cells on a HA-coated genipin-chitosan scaffold *in vitro* was studied followed by evaluation on bone repair *in vivo* [903]. The study demonstrated the potential of this formulation for bone regeneration.

## 9. Conclusions and Outlook

The available chronology of seeking for a suitable bioceramics for bone substitutes is as follows: since the 1950s, the first aim was to use bioinert bioceramics, which had no reaction with living tissues. They included inert and tolerant compounds, which were designed to withstand physiological stress without, however, stimulating any specific cellular responses. Later on, in the 1980s, the trend changed towards exactly the opposite: the idea was to implant bioceramics that reacted with the surrounding tissues by producing newly formed bone (a “responsive” bioceramics because it was able to elicit biological responses). These two stages have been referred to as the first and the second generations of bioceramics, respectively [904] and, currently, both of them have been extensively commercialized. Thus, the majority of the marketable products listed in Table 3 belong to the first and the second generations of bone substitute biomaterials. However, the progress keeps going and, in current century, scientists search for the third generation of bioceramics [327], which will be able to “instruct” the physiological environment toward desired biological responses (*i.e.*, bioceramics will be able to regenerate bone tissues by stimulating specific responses at the molecular level) [60,62]. Since each generation represents an evolution on the requirements and properties of the biomaterials involved, one should stress that these three generations should not be interpreted as the chronological but the conceptual ones. This means that at present, research and development is still devoted to biomaterials and bioceramics that, according to their properties, could be considered to be of the first or the second generations, because the second generation of bioceramics with added porosity is one of the initial approaches in developing of the third generation of bioceramics [905]. Furthermore, there is another classification of the history of biomaterials introduced by Prof. James M. Anderson. According to Anderson, within 1950–1975 the researchers studied bioMATERIALS, within 1975–2000 they studied BIOMATERIALS and since 2000 the time for BIOmaterials has been coming [906]. Here, the capital letters emphasis the major direction of the research efforts in the complex subject of biomaterials. As bioceramics are biomaterials of the ceramic origin (see Section 2), the Anderson’s historical classification appears to be applicable to the bioceramics field as well.

The history development of bioceramics, one category of biomaterials, informs that the widespread use of biomaterials, however, experiences two major difficulties. The first is an incomplete understanding of the physical and chemical functioning of biomaterials and of the human response to these materials. Recent advances in material characterization and computer science, as well as in cell and molecular biology are expected to play a significant role in studies of biomaterials. A second difficulty is that many biomaterials do not perform as desirably as we would like. This is not surprising, since many materials used in medicine were not designed for medical purposes. It needs to be mentioned here that biomaterials are expected to perform in our body’s internal environment, which is very aggressive. For example, solution pH of body fluids in various tissues varies in the range from 1 to 9. During daily activities, bones are subjected to a stress of ~4 MPa, whereas the tendons and ligaments experience peak stresses in the range of 40–80 MPa. The mean load on a hip joint is up to three times body weight (3000 N) and peak load during jumping can be as high as ~10 times body weight. More importantly, these stresses are repetitive and fluctuating, depending on the activities, such as standing, sitting, jogging, stretching and climbing. All of these require careful designing of biomaterials in terms of composition, shape, physical and biocompatibility properties. Therefore, a significant challenge is the rational design of human biomaterials based on a systematic evaluation of desired biological, chemical and engineering requirements.

Nevertheless, the field of biomaterials is in the midst of a revolutionary change in which the life sciences are becoming equal in importance to materials science and engineering as the foundation of the field. Simultaneously, advances in engineering (for example nanotechnology) are greatly increasing the sophistication with which biomaterials are designed and have allowed fabrication of biomaterials with increasingly complex functions [907]. Specifically, during last ~40 years, calcium orthophosphate bioceramics has become an integral and vital segment of our modern health care delivery system. In the modern fields of the third generation bioceramics (Hench) or BIOceramics (Anderson), the full potential of calcium orthophosphates has only begun to be recognized. Namely, calcium orthophosphates, which were intended as osteoconductive bioceramics in the past, stand for materials to fabricate osteoinductive implants nowadays [171,563,592,666,667,668,669,670,671,672,673,674,675,676,677,678,679,680,681,682,683,684,685]. Some steps in this direction have been already made by fabricating scaffolds for bone tissue engineering through the design of controlled 3D-porous structures and increasing the biological activity through development of novel ion-substituted calcium orthophosphate bioceramics [28,565]. The future of biosynthetic bone implants will point to better mimicking the autologous bone grafts. Therefore, the composition, structure and molecular surface chemistry of various types of calcium orthophosphates will be tailored to match the specific biological and metabolic requirements of tissues or disease states [908,909]. This new generation of calcium orthophosphate bioceramics should enhance the quality of life of millions of people, as they grow older.

In spite of the great progress, there is still a great potential for major advances to be made in the field of calcium orthophosphate bioceramics [910]. This includes requirements for:

1. Improvement of the mechanical performance of existing types of bioceramics.
2. Enhanced bioactivity in terms of gene activation.
3. Improvement in the performance of biomedical coatings in terms of their mechanical stability and ability to deliver biological agents.
4. Development of smart biomaterials capable of combining sensing with bioactivity.
5. Development of improved biomimetic composites.

Furthermore, still there are needs for a better understanding of the biological systems. For example, the bonding mechanism between the bone mineral and collagen remains unclear. It is also unclear whether a rapid repair that is elicited by the new generation of bioceramics is a result of the enhancement of mineralization *per se* or whether there is a more complex signaling process involving proteins in collagen. If we were able to understand the fundamentals of bone response to specific ions and the signals they activate, then we would be able to design better bioceramics for the future [910].

To finalize this review, it is completely obvious that the present status of research and development in the field of calcium orthophosphate bioceramics is still at the starting point for the solution of new problems at the confluence of materials science, biology and medicine, concerned with the restoration of damaged functions in the human organisms. A large increase in active elderly people has dramatically raised the need for load-bearing bone graft substitutes, for example, for bone reconstruction during revision arthroplasty or for the reinforcement of osteoporotic bones. Strategies applied in the last four decades towards this goal have failed. So new strategies, possibly based on self-assembling and/or nanofabrication, will have to be proposed and developed [911]. Furthermore, in future, it should be feasible to design a new generation of gene-activating calcium orthophosphate based scaffolds tailored for specific patients and disease states. Perhaps, sometime bioactive stimuli will be used to activate genes in a preventative treatment to maintain the health of aging tissues. Currently this concept seems impossible. However, we need to remember that only ~40 years ago the concept of a material that would not be rejected by living tissues also seemed impossible [654].
